# A novel mechanism of RNase L inhibition: Theiler's virus L* protein prevents 2-5A from binding to RNase L

**DOI:** 10.1371/journal.ppat.1006989

**Published:** 2018-04-13

**Authors:** Melissa Drappier, Babal Kant Jha, Sasha Stone, Ruth Elliott, Rong Zhang, Didier Vertommen, Susan R. Weiss, Robert H. Silverman, Thomas Michiels

**Affiliations:** 1 Université catholique de Louvain, de Duve Institute, Brussels, Belgium; 2 Translational Hematology and Oncology Research, Taussig Cancer Research Institute, Cleveland Clinic, Cleveland, Ohio, United States of America; 3 Department of Microbiology, Perelman School of Medicine at the University of Pennsylvania, Philadelphia, Pennsylvania, United States of America; 4 Department of Cancer Biology, Lerner Research Institute, Cleveland Clinic, Cleveland, Ohio, United States of America; NYU School of Medicine, UNITED STATES

## Abstract

The OAS/RNase L pathway is one of the best-characterized effector pathways of the IFN antiviral response. It inhibits the replication of many viruses and ultimately promotes apoptosis of infected cells, contributing to the control of virus spread. However, viruses have evolved a range of escape strategies that act against different steps in the pathway. Here we unraveled a novel escape strategy involving Theiler’s murine encephalomyelitis virus (TMEV) L* protein. Previously we found that L* was the first viral protein binding directly RNase L. Our current data show that L* binds the ankyrin repeats R1 and R2 of RNase L and inhibits 2’-5’ oligoadenylates (2-5A) binding to RNase L. Thereby, L* prevents dimerization and oligomerization of RNase L in response to 2-5A. Using chimeric mouse hepatitis virus (MHV) expressing TMEV L*, we showed that L* efficiently inhibits RNase L *in vivo*. Interestingly, those data show that L* can functionally substitute for the MHV-encoded phosphodiesterase ns2, which acts upstream of L* in the OAS/RNase L pathway, by degrading 2-5A.

## Author summary

Oligoadenylate synthetases (OAS) are a family of enzymes that produce 2’-5’ oligoadenylates (2-5A) from ATP. OAS accumulate as inactive enzymes in cells that have been exposed to interferon. When infection of these cells occurs, double-stranded RNA produced during virus replication activates OAS, which produce 2-5A. 2-5A act as second messengers. They bind a cellular endonuclease called RNase L, thereby triggering the dimerization and the activation of this RNase. Active RNase L degrades cellular and viral RNA molecules, ultimately killing the infected cell, thus preventing virus spread. Viruses have developed diverse mechanisms to escape RNase L activity. We report that Theiler’s murine encephalomyelitis virus, a virus that produces persistent infections of the central nervous system, evolved a novel mechanism to escape the OAS/RNase L pathway. The L* protein produced by this virus binds RNase L very close to the pocket known to sense 2-5A and thereby prevents 2-5A from activating RNase L. This mechanism contrasts with that used by Influenza (flu) virus, which sequesters double-stranded RNA and thereby also inhibits alternative antiviral pathways. We further showed that the RNase L antagonist activity of L* is effective *in vivo* and that L* can substitute for 2-5A degrading enzymes produced by coronaviruses.

## Introduction

Theiler’s murine encephalomyelitis virus (TMEV or Theiler’s virus) belongs to the genus *Cardiovirus* in the family *Picornaviridae*. Persistent strains of TMEV, such as DA, BeAn or TO4 have a remarkable ability to establish persistent infections of the mouse central nervous system. In susceptible mouse strains, TMEV can persist lifelong, replicating mainly in oligodendrocytes, macrophages and microglial cells, in the white matter of the spinal cord [1–5]. Two accessory proteins, L and L*, were shown to contribute to the ability of TMEV to establish persistent infections by interfering with the host innate immune response [2, 5]. The L* (read “L-star”) protein is an 18kDa protein encoded by an alternative open reading frame overlapping the L-, VP4- and VP2-coding regions of the ORF encoding the viral polyprotein [6]. It facilitates the infection of macrophages *in vitro* [7, 8] and the initiation of persistent infections *in vivo* [9, 10]. L* is first detected in the cytosol of infected cells and subsequently accumulates in the mitochondrial outer membrane [11]. We previously showed that L* can inhibit RNase L through direct protein-protein interaction [12]. Interestingly, RNase L inhibition by L* is a highly species-specific process. Indeed, L* of the mouse TMEV strain DA inhibits mouse RNase L but not its orthologues from other tested species including rat [12], whereas L* of the rat TMEV strain RTV-1 inhibits rat but not human or even mouse RNase L [13].

RNase L is the effector enzyme of the OAS/RNase L system, one of the best-characterized interferon-induced antiviral pathways (recently reviewed in [14, 15]). Interferon secreted in response to viral infection induces the expression of a family of enzymes: the oligoadenylate synthetases (OAS). OAS are produced as inactive enzymes and become activated upon binding dsRNA, a byproduct of viral replication. Active OAS catalyze the conversion of ATP into 2’-5’ oligoadenylates called 2-5A [16–19]. 2-5A then act as second messengers and bind to monomeric inactive RNase L, triggering its dimerization and its activation [20]. Enzymatically active RNase L cleaves viral and cellular single-stranded RNA and therefore inhibits protein synthesis, decreases viral replication, and ultimately leads to apoptosis [19, 21, 22]. Through the generation of short RNA fragments, RNase L can further amplify IFN signaling [23]. RNase L products were also reported to trigger autophagy and to activate the inflammasome [24–26].

RNase L is composed of three domains: an N-terminal ankyrin domain formed of 9 ankyrin repeats (ANK R1-9) involved in 2-5A recognition, a central catalytically inactive pseudokinase domain contributing to RNase L dimerization, and a C-terminal ribonuclease domain responsible for target RNA cleavage [27]. In the active, dimeric RNase L, two 2-5A molecules are embedded in a composite pocket formed by ANK R2 and ANK R4 of one protomer, and ANK R9 and the pseudokinase N-terminal lobe of the other protomer [27–30]. Structural analysis of human RNase L suggested that the enzyme could form higher-order oligomers whose activity would be increased compared to that of dimers [29]. Oligomer formation was however not observed in structural studies of *Sus scrofa* RNase L [30].

RNase L is active against many viruses such as West Nile virus [31] Encephalomyocarditis virus (EMCV) [21], Coxsackievirus B4 [32] and Mouse hepatitis virus (MHV) [33]. As a consequence, viruses have evolved several strategies to escape this pathway. Interestingly, almost any step of the OAS/RNase L pathway can be targeted by viruses (for review: [34, 35]). For instance, Influenza A virus encodes the NS1 protein that sequesters dsRNA, thereby preventing OAS activation [36]. More recently, it was shown that MHV, Middle East Respiratory Syndrome coronavirus (MERS-CoV) and rotaviruses express the phosphodiesterases, ns2, NS4b, and VP3 respectively, which degrade 2-5A and thus prevent RNase L activation [33, 37, 38]. Poliovirus acts downstream of the pathway through a highly structured genomic RNA region, which acts as a cleavage-resistant substrate for RNase L [39, 40]. As mentioned above, we recently observed that TMEV L* directly binds RNase L suggesting a novel mechanism of RNase L inhibition [12]. As L* was found to bind the N-terminal part of RNase L (residues 1–400) encompassing the entire ankyrin domain (ANK R1-R9), L* most likely acts by inhibiting 2-5A binding to RNase L, or by preventing RNase L dimerization. It is however not excluded that L* could act in an allosteric fashion to block the catalytic activity of RNase L.

This study aimed at unraveling the novel mechanism of RNase L inhibition involving TMEV L*. Our results show that L* binds RNase L ankyrin repeats very close to 2-5A binding pockets, thereby inhibiting 2-5A binding to RNase L. Using chimeric viruses, we further provide evidence that L* can substitute for another viral RNase L inhibitor acting upstream of L* in the OAS/RNase L cascade, namely the ns2 phosphodiesterase encoded by MHV.

## Results

### RNase L ANK repeats 1 and 2 are sufficient for L* binding

**Fig 1:**
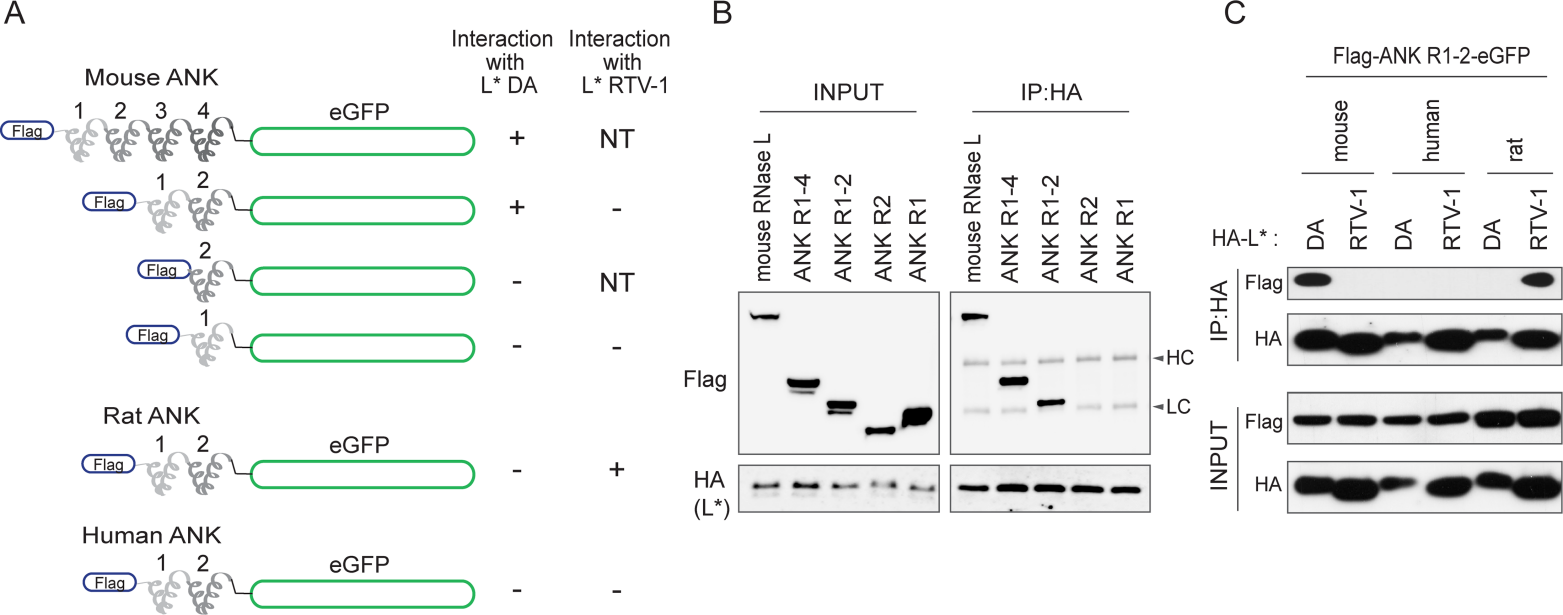
RNase L ANK repeats 1 and 2 are sufficient for interaction with L*. A. Enhanced Green Fluorescent Protein (eGFP) was fused to the C-terminus of indicated Flag-tagged ankyrin repeats of mouse, rat and human RNase L. results of L* co-immunoprecipitation with eGFP constructs shown in B and C are summarized. NT: not tested. B. Co-immunoprecipitation of indicated Flag-tagged eGFP fusion proteins with HA-tagged L*DA. Right panels show Flag (ANK repeats) and HA (L*) detection after immunoprecipitation of HA. Left panels show detection of Flag and HA in cell lysates (Input). Similar results were obtained in 2 independent experiments. HC: immunoglobulin heavy chain, LC: immunoglobulin light chain. C. Co-immunoprecipitation of Flag-ANK R1-2-eGFP from indicated species with HA-tagged L* of DA or RTV-1. Upper panels show Flag and HA (L*) detection after immunoprecipitation of HA. Lower panels show detection of Flag and HA in cell lysates (Input). Shown are results representative of 2 independent experiments.

We previously showed that L* binds the ankyrin domain of RNase L. To identify the region within the ankyrin domain that is targeted by L*, enhanced green fluorescent protein (eGFP) fusions were constructed that carry ANK repeats of mouse, rat and human RNase L upstream of the eGFP coding sequence (Fig 1A). The ANK-eGFP fusion proteins were N-terminally Flag-tagged. We next tested whether ANK-eGFP fusion co-immunoprecipitated with HA-tagged L* (HA-L*), in extracts of transfected 293T cells. As summarized in Fig 1A, L*_DA_ interacted with fusion proteins carrying mouse RNase L ANK R1-R2 but not with fusions carrying ANK R2 or ANK R1 alone (Fig 1B). Specificity of the interaction was confirmed by showing that L*_DA_ only interacted with mouse ANK R1-R2 constructs while L*_RTV-1_ only interacted with rat ANK R1-R2 (Fig 1C). We concluded that ankyrin repeats 1 and 2 of mouse and rat RNase L are sufficient for binding of L*_DA_ and L*_RTV-1_ respectively.

In infected cells, L* is both cytosolic and partly anchored in the mitochondrial outer membrane [11]. Using the Flag-muANK R1-2-eGFP construct that readily interacts with L*_DA_, we observed by fluorescent microscopy that the soluble pool of cytosolic L* was responsible for RNase L binding (S1 Fig).

### Human/Mouse RNase L chimeras define a region in ANK R1 as a site required for L* binding

**Fig 2:**
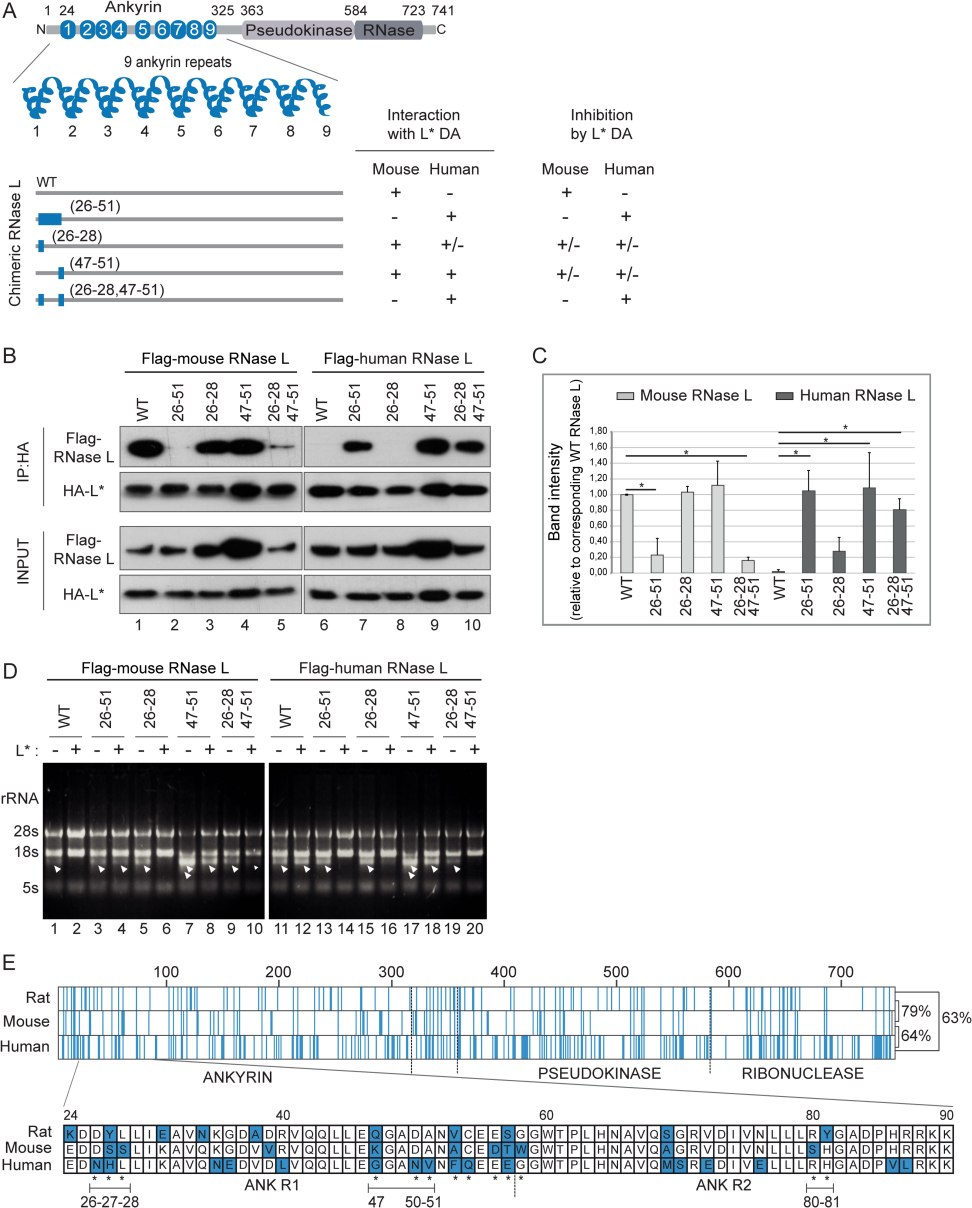
Amino acids 26 to 51 of the mouse RNase L are required for interaction with and inhibition by L*. A. The structure of the ankyrin domain of tested mouse/human RNase L chimeras is presented under a cartoon showing the general organization of RNase L. Blue thick lines (with numbered residues) represent the RNase L segments within the ankyrin domain (to scale with the upper cartoon) that were exchanged between human and mouse RNase L. The table given right of the cartoon summarizes binding and functional data obtained with symmetrical mouse and human RNase L chimeras. B-C. Co-immunoprecipitation of indicated Flag-RNase L (left: mouse RNase L, right: human RNase L) with HA-L*_DA_. Immunoblots (B) show Flag and HA detection after immunoprecipitation of HA-L* (IP:HA) and in cell lysates (Input). Graphs (C) show the mean and SD of the amount of co-immunoprecipitated RNase L chimera relative to that of WT mouse RNase L (n = 3). *: p<0.05 in a two-way ANOVA followed by Dunnett’s test for multiple comparison. D. Analysis of RNase L-mediated RNA degradation in HeLa-M cells overexpressing indicated Flag-RNase L and L*_DA_. RNA samples were extracted 7 hours after polyI:C transfection. Arrowheads point to rRNA cleavage products. Reproducible results were obtained in 2 independent experiments. E. Alignment of rat, mouse and human RNase L protein sequences. The upper part shows a schematic representation of the alignment. Blue lines represent amino acids that differ from the two other sequences. Percentages of sequence identity are indicated on the right of the alignment. The lower part shows a zoom in ANK R1 and R2 amino acid sequences. Blue residues are residues that differ from the two other sequences. Asterisks indicate amino acids that were tested in the chimeric constructs. Underlined sequences correspond to those affecting L* binding.

To define whether L* interacts with 2-5A binding sites or with dimerization interfaces of RNase L, we mapped the footprint of L* on RNase L, taking advantage of the exquisite species-specificity of RNase L antagonism by L*. Mouse and human RNase L share 64% amino-acid sequence identity, divergent residues being spread throughout the protein (Fig 2). We thus tested the ability of L* from the mouse virus strain DA to bind and inhibit a series of chimeras constructed between mouse and human RNase L (Fig 2A–2D). Binding was assessed by co-immunoprecipitation of N-terminally Flag-tagged RNase L chimeras (Flag-RNase L) with HA-tagged L* (HA-L*)(Fig 2A–2C). As expected, L* interacted with mouse but not with human RNase L (Fig 2B lane 1, 6). Interestingly, swapping either the first RNase L ANK repeat (residues 1–58) or even residues 26 to 51 within this repeat (Fig 2B, lanes 2 and 7) led to a complete swap of L* binding abilities. Within this segment, several amino acid stretches diverge between human and mouse RNase L (Fig 2E). Exchanging residues 26–28 did not lead to L* binding to human RNase L (Fig 2B, lane 8). Exchanging residues 47–51 led to recognition of both human and mouse RNase L by L* (Fig 2B, lanes 4 and 9). Exchanging both residue stretches led to a significant swap in L* binding abilities as L* readily bound human RNase L (Fig 2B, lane 10) and almost completely lost the ability to bind mouse RNase L (Fig 2B, lane 5) (Fig 2C). Thus, amino acid stretches D26, S27, S28 and K47, D50, A51 within ANK R1 of mouse RNase L are important for L* binding.

To confirm the binding data, we tested whether L* similarly affected RNase activity of the human/mouse RNase L chimeras. For this purpose, constructs expressing Flag-RNase L chimeras and constructs co-expressing L* and mCherry were transfected in HeLa-M cells, which show minimal endogenous RNase L activity [41]. The OAS/RNase L pathway was activated in these cells by transfecting polyinosinic:polycytidylic acid (polyI:C). RNA was then extracted and separated on agarose gel and on RNA chips (Fig 2D and S2A Fig) to evaluate RNA degradation (i.e. RNase L activity). Again, mouse RNase L was inhibited by L* whereas human RNase L was not (Fig 2D and S2A Fig, lanes 2 and 12), and swapping residues 26–51 of ANK R1 resulted in inhibition of human but not mouse RNase L by L* (lanes 4 and 14). Swapping residues 26–28 and 47–51 led to a clear inhibition of human RNase L while decreasing L* influence on mouse RNase L activity (lanes 10 and 20).

In general, results from interaction and functional studies were congruent. However, for some constructs such as mouse RNase L G47N50V51, interaction with L* was conserved while activity inhibition was weak (Fig 2B–2D). A possible explanation for such discrepancies is that L* still binds to this chimera but with an affinity that is too low to trigger RNase L inhibition.

Taken together, these data show that residue stretches D26, S27, S28 and K47, D50, A51 within ankyrin repeat 1 of mouse RNase L contribute to L* binding to RNase L and to L*-mediated RNase L inhibition.

### A motif in ANK R2 contributes to L* binding

**Fig 3:**
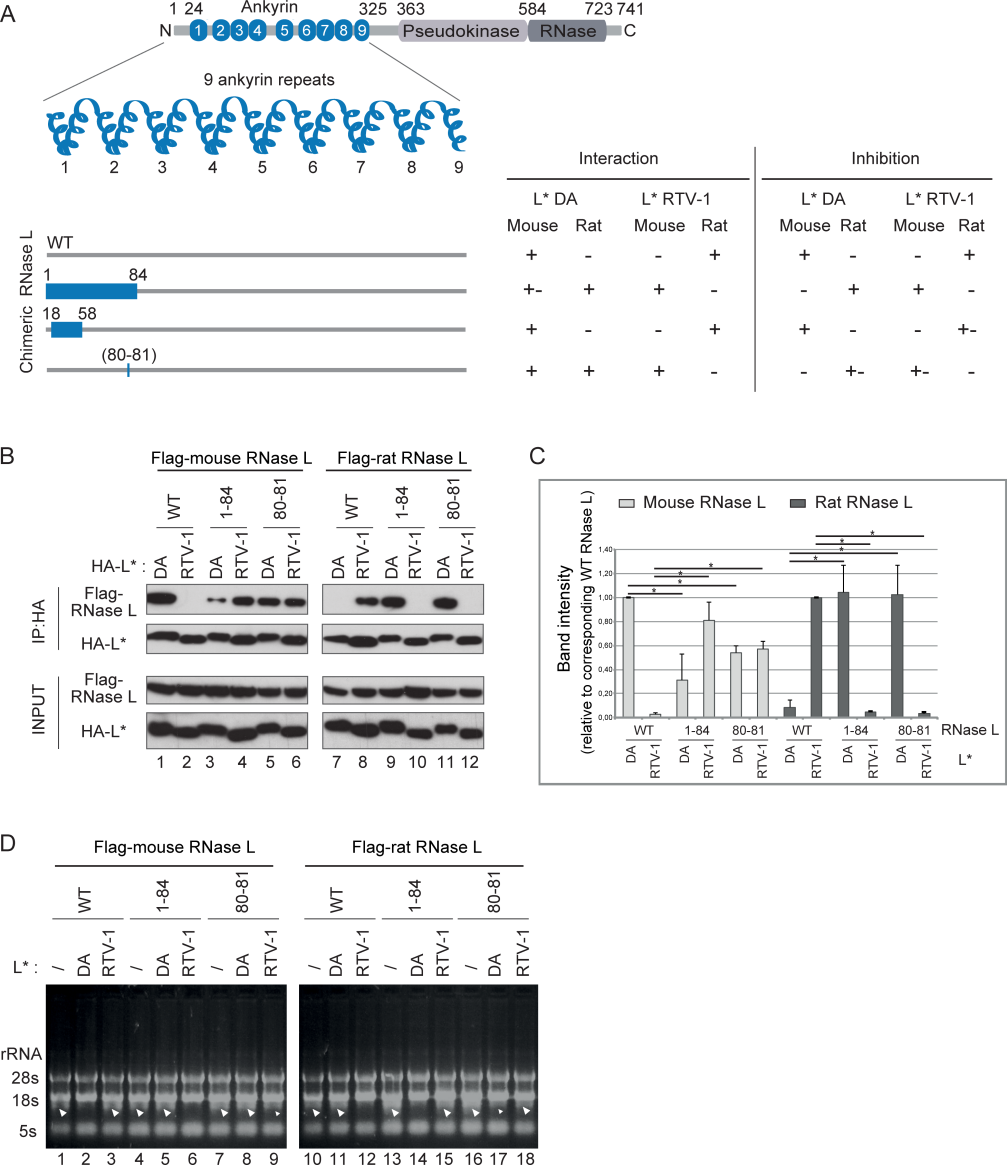
Amino acids 81 and 82 of the mouse RNase L are also necessary for interaction with and inhibition by L*. A. Segments of decreasing size were swapped between rat and mouse RNase L and the resulting chimeric RNase L were tested for inhibition by and interaction with L*_DA_ (mouse virus) and L*_RTV-1_ (rat virus)_._ Schematic rat/mouse chimeric RNase L are represented as in Fig 2. The right columns indicate whether L*_DA_ and L*_RTV-1_ interact with and inhibit the chimeric enzymes. B-C. Immunoblots (B) show Flag and HA detection after immunoprecipitation of HA-L* (IP:HA) and in cell lysates (Input). Graphs (C) show the mean and SD of the amount of co-immunoprecipitated RNase L chimera relative to that of WT RNase L of the corresponding species (n = 3). *: p<0.05 in a two-way ANOVA followed by Dunnett’s test for multiple comparison. D. Analysis of RNase L-mediated RNA degradation in HeLa-M cells overexpressing indicated Flag-RNase L and L*_DA_ or L*_RTV-1_. RNA samples were extracted 7 hours after polyI:C transfection. Arrowheads point to rRNA cleavage products. Reproducible results were obtained in 2 independent experiments.

The use of human/mouse RNase L chimeras does not allow identification of L* binding residues that are conserved between the RNase L proteins of the two species. Since we recently cloned rat RNase L and showed that L* from the rat TMEV strain RTV-1 inhibited rat but not mouse RNase L, we extended the analysis to rat/mouse RNase L chimeras (Fig 3A). Rat and mouse RNase L share 79% amino acid identity (Fig 2E). In this case, L* binding (Fig 3B and 3C) and L*-mediated RNase L inhibition (Fig 3D) were assessed using both L* of the mouse TMEV strain DA (L*_DA_) and L* of the rat TMEV strain RTV-1 (L*_RTV-1_).

As expected, mouse RNase L interacted with and was inhibited by L*_DA_ but not L*_RTV-1_, while rat RNase L was inhibited by and interacted with L*_RTV-1_ but not L*_DA_ (Fig 3B, lanes 1, 2, 7, 8; Figs 3D and S2B, lanes 2, 3, 11, 12). Swapping ANK R1-R2 (residues 1–84) was sufficient to swap both interaction and inhibition phenotypes (Fig 3B and 3C, lanes 3, 4, 9, 10; Figs 3D and S2B, lanes 5, 6, 14, 15). In contrast to what was observed with human/mouse chimeras, swapping ANK R1 (residues 18–58) alone was not sufficient to exchange the phenotypes, indicating that residues in ANK R2 were also required for L* interaction. Insertion of point mutations based on differences between rat and mouse sequences showed that swapping residues 80–81 in ANK R2 was sufficient to trigger rat RNase L recognition by L*_DA_ (Fig 3B, compare lanes 7 and 11; Fig 3D, compare lanes 11 and 17) and mouse RNase L recognition by L*RTV-1 (Fig 3B, compare lanes 2 and 6; Fig 3D, compare lanes 3 and 9). Although the exchange of phenotype was not complete (i.e. some interaction was still detected between L*_DA_ and mouse RNase L^R80,Y81^), these data show that residues 80 and 81 of ANK R2 contribute to L* binding to RNase L and L*-mediated RNase L inhibition.

**Fig 4:**
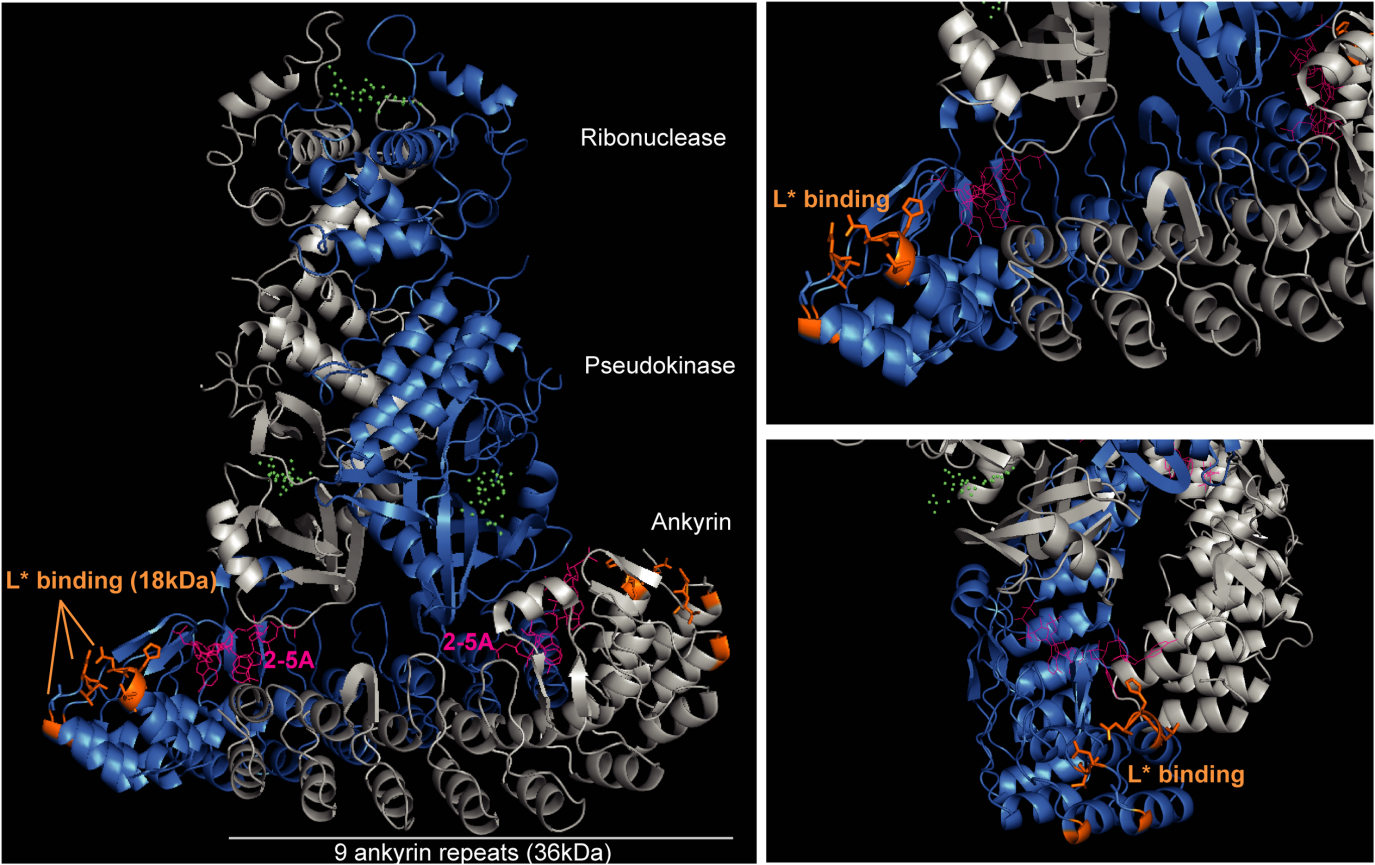
L* footprint on RNase L. Highlighting of residues that were mapped in ANK R1 and R2 as components of the L* footprint on RNase L (in orange). The two RNase L protomers are represented in gray and blue, 2-5A molecules are represented in pink and RNA substrate and ATP mimetics are represented by green dots. (Crystal structure of dimeric human RNase L; [45]; PDB 4OAV). Images were generated using the PyMOL Molecular Graphics System, Version 1.7, Schrödinger, LLC.

Fig 4 illustrates residues that were mapped in ANK R1 and R2 as components of the L* footprint on RNase L. Being located very close to both a 2-5A binding site and a protomer-protomer interface, the L* interaction site does not resolve the question of whether L* interferes with 2-5A binding or with RNase L dimer formation.

### RNase L residues involved in 2-5A binding are not targeted by L*

**Fig 5:**
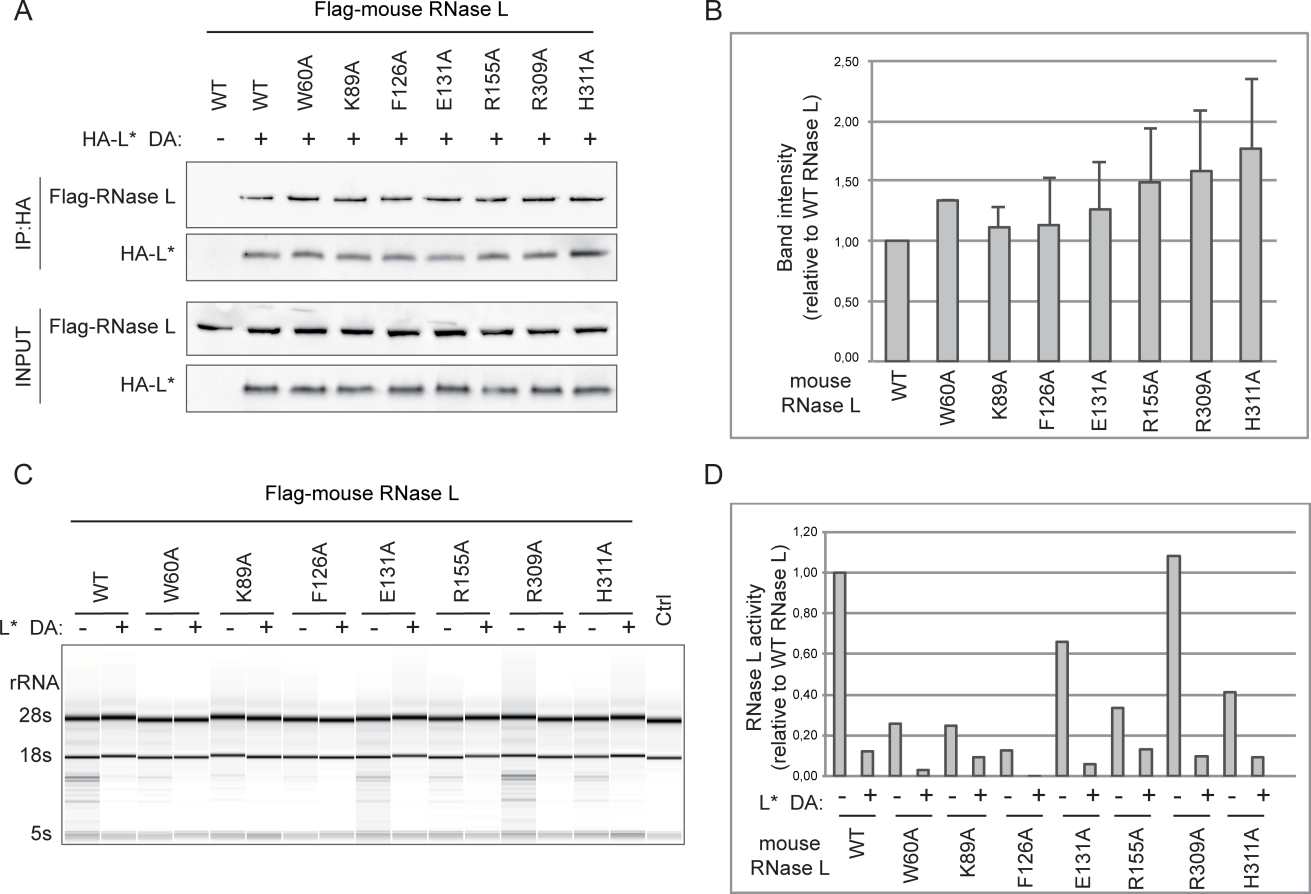
RNase L residues involved in 2-5A binding are not crucial for L* binding to mouse RNase L. A-B. Co-immunoprecipitation of indicated 2-5A binding-defective mouse RNase L mutants with HA-tagged L*DA. A. Immunoblots show Flag (RNase L) and HA (L*) detection after immunoprecipitation of HA (upper panels) and in cell lysates (Input, lower panels). B. Graphs showing the quantification of coimmunoprecipitated RNase L mutants relative to coimmunoprecipitated WT mouse RNase L (n = 3). Differences were non-significant according to one-way ANOVA followed by Tukey's test for multiple comparisons. C-D. Analysis of RNase L-mediated RNA degradation in HeLa-M cells overexpressing indicated Flag-RNase L and HA-L*_DA_. RNA samples extracted 7 hours after polyI:C transfection were analyzed by RNA chips (C) and quantified (D). Graphs show the quantification of RNA degradation by RNase L mutants in the absence or in the presence of L*_DA_. Data are normalized to those of WT RNase L in the absence of L*.

Structural and functional studies identified human RNase L residues that are involved in 2-5A binding [28, 29, 42]. Because most of these residues are conserved across species, the chimeric RNase L strategy did not allow to test how much these residues were targeted by L*. We thus introduced point mutations in Flag-mouse RNase L constructs, that were shown to inhibit 2-5A binding in the case of human RNase L. This included mutations W60A and K89A of ANK R2 as well as mutations in ANK R4 and ANK R9 that were analyzed as controls. As shown in Fig 5A and 5B, none of the tested mutation significantly affected HA-L* binding to RNase L, suggesting that L* binds close to but not at the sites of 2-5A binding. Functional tests were performed as above to assess the capacity of mutant RNase L to degrade RNA (Fig 5C and 5D). Surprisingly, RNase L activity was not affected by mutation R309A and only partly affected by mutations E131A, H311A or R155A, suggesting that residues involved in 2-5A binding partly vary between mouse and human RNase L.

### L* inhibits mouse RNase L dimerization and oligomerization

We next established an RNase L dimerization assay based on disuccinimidylsuberate (DSS)-mediated crosslinking. 293T cells overexpressing a C-terminally Flag-tagged RNase L (RNase L-Flag) and L* were gently lysed. Then, 2-5A molecules were added to the lysate to promote RNase L dimerization before addition of DSS. After incubation, a fraction of the sample was used for RNA extraction to monitor 2-5A-dependent (i.e. RNase L-mediated) RNA degradation. The remaining part of the lysate was used to monitor RNase L dimerization by immunoblotting (20).

**Fig 6:**
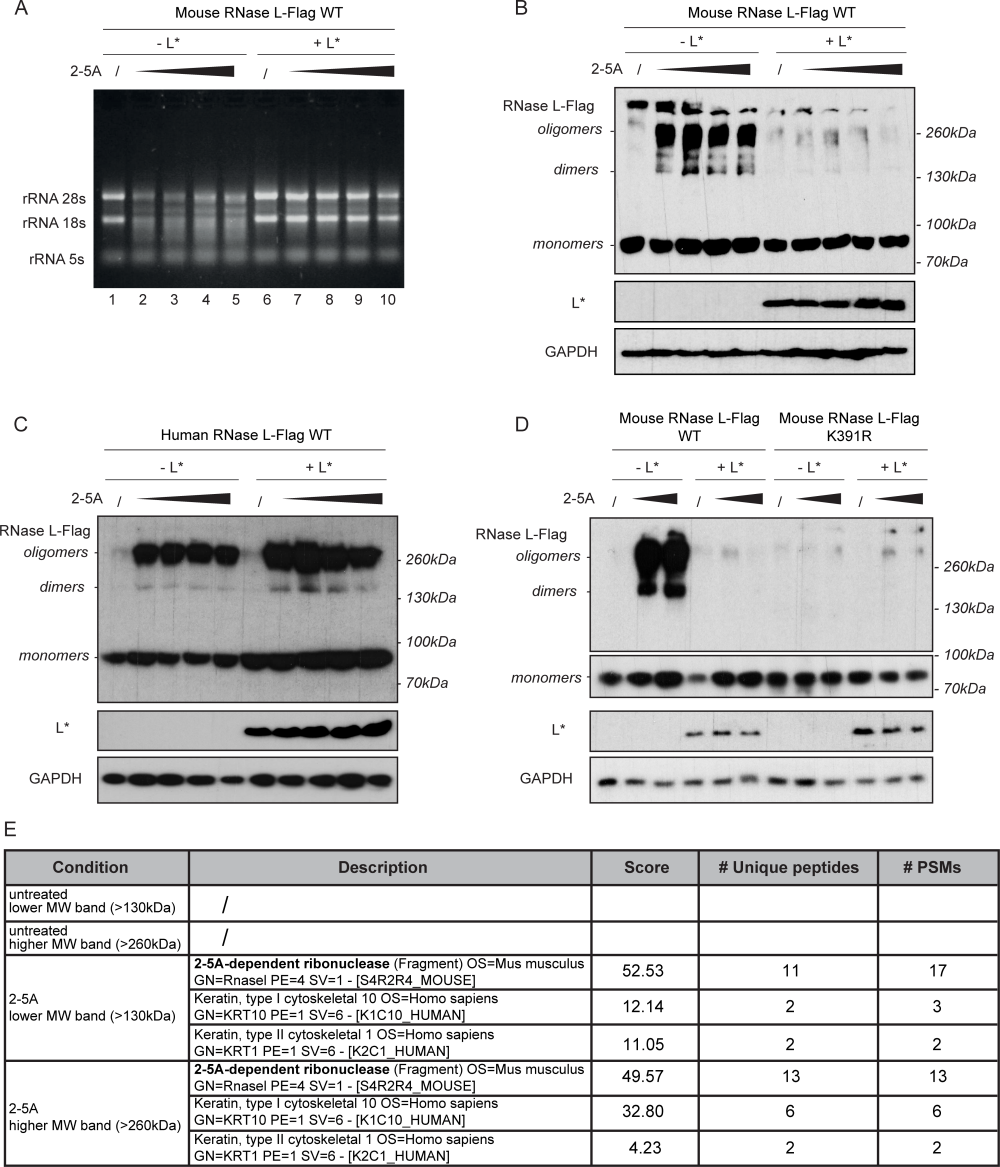
L* inhibits mouse RNase L dimerization and oligomerization. C-terminal Flag-tagged RNase L (RNase L-Flag) and L* were overexpressed in 293T cells by transfection. Cells lysates were divided into samples where RNase L dimerization was induced by adding increasing concentrations of 2-5A. Dimers/oligomers were crosslinked using DSS. A. RNA was extracted from lysates (same samples as in B) and analyzed on agarose gel electrophoresis as a control for RNase L activation. 2-5A concentrations: 0.25, 0.5, 1 and 2μM ATP equivalent. B. The upper panel shows the detection by immunoblot of WT mouse RNase L-Flag monomers, dimers and oligomers using an anti-Flag antibody. Lower panels show detection of L* and GAPDH used as a loading control. 2-5A concentrations: 0.25, 0.5, 1 and 2μM ATP equivalent. C. The upper panel shows the detection of WT human RNase L-Flag monomers, dimers and oligomers using an anti-Flag antibody. Lower panels show detection of L* and GAPDH. 2-5A concentrations: 0, 2, 4 and 8μM ATP equivalent. D. Dimerization-defective (K391R) mouse RNase L was used as a dimerization control. The upper panel shows the detection of RNase L-Flag monomers, dimers and oligomers using an anti-Flag antibody. Lower panels show detection of L* and GAPDH. 2-5A concentrations: 0.5 and 2μM ATP equivalent. Reproducible results were obtained in 2 independent experiments. E. Mass spectrometry analysis of high molecular weight RNase L complexes. Proteins for which 2 or more unique peptides were detected, with a false discovery rate below 5%, are listed in the table. # PSMs: number of peptide spectrum matches. /: no peptide detected above threshold.

As can be seen in Fig 6, in the absence of L*, rRNA was degraded in a dose-dependent manner upon 2-5A treatment, demonstrating RNase L activation (Fig 6A, lanes 1–5). In the presence of L*, rRNA integrity was preserved (Fig 6A, lanes 6–10), confirming that L* could inhibit RNase L in the conditions used for the crosslinking experiment. Immunoblotting showed that RNase L formed dimers and oligomers when activated by 2-5A (Fig 6B). The corresponding bands were much less intense when L* was expressed, showing that L* inhibits mouse RNase L dimerization and oligomerization. When the experiment was reproduced with human RNase L, the appearance of high molecular weight bands upon 2-5A addition was not inhibited by L*, as expected (Fig 6C). To further confirm the identity of RNase L dimers and oligomers, the experiment was repeated using a dimerization-defective mouse RNase L mutant (RNase L K391R) [43]. High molecular weight bands observed with the wild-type RNase L upon 2-5A treatment were not obtained with the dimerization-defective mutant (Fig 6D), confirming that these bands indeed corresponded to RNase L dimers and oligomers.

We next analyzed whether oligomers formed in the presence of 2-5A involved additional protein partners or whether they resulted from homooligomerization. Therefore, crosslinking was performed after 2-5A addition as above, using the reversible agent dithiobis(sulfosuccinimidyl propionate) (DTSSP) that is compatible with further mass spectrometry analysis. High molecular weight complexes were then separated by SDS-PAGE and analyzed by mass spectrometry (Fig 6E). Except for keratin which is a typical contaminant, no specific protein other than RNase L was detected in the complexes, suggesting that RNase L oligomers corresponded to homooligomers.

Collectively, these data show that 2-5A can trigger the dimerization and the homooligomerization of RNase L and that L* binding to RNase L inhibits these processes.

### L* interferes with 2-5A binding to mouse RNase L

**Fig 7:**
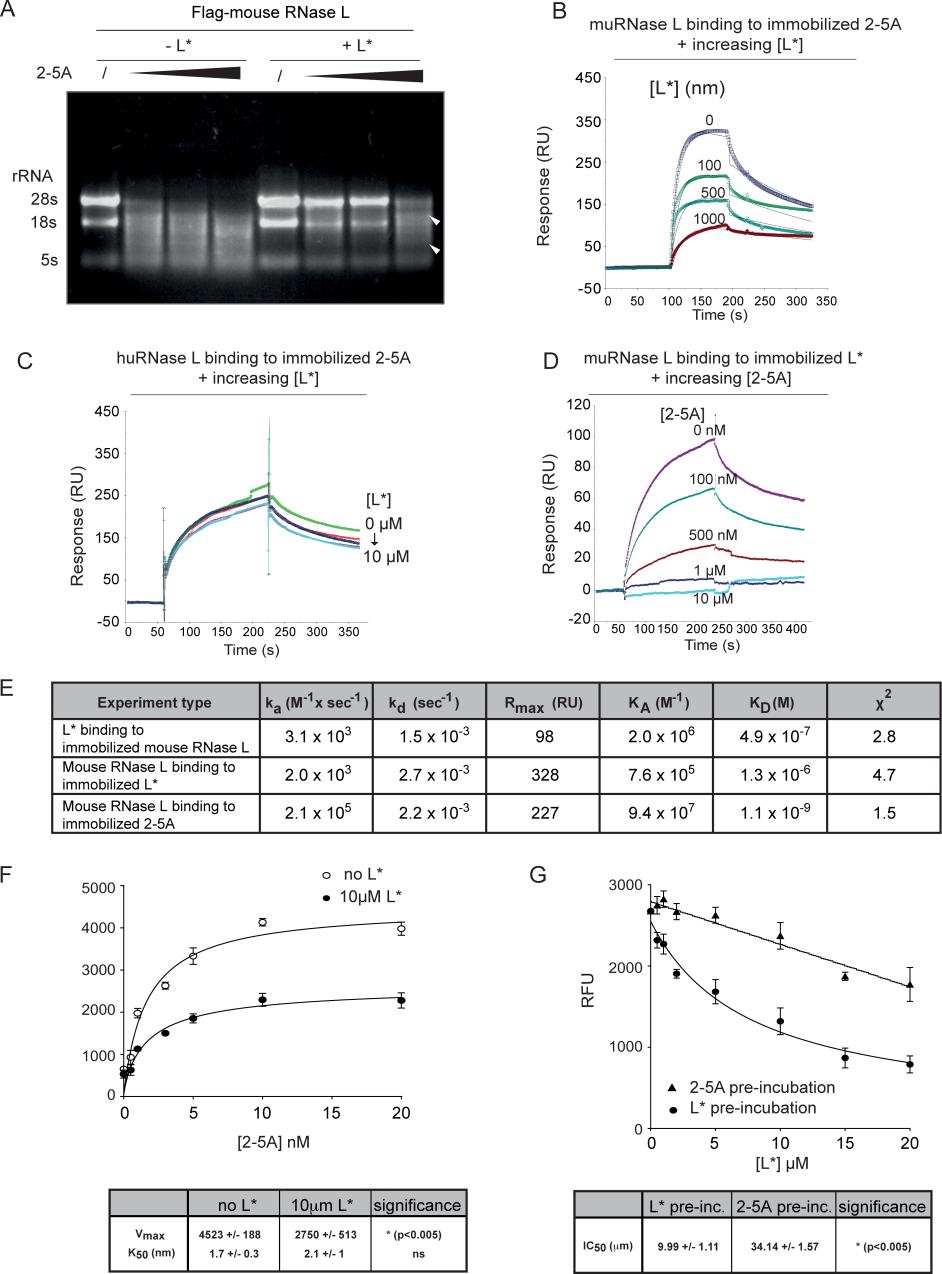
L* interferes with 2-5A binding to mouse RNase L. A. Lysates of 293T cells overexpressing Flag-RNase L and L* were treated with increasing concentrations of 2-5A (0, 2, 4 and 8 μM ATP equivalent of 2-5A) before incubation, RNA extraction and analysis on agarose gel. Arrowheads indicate RNase L-generated rRNA degradation products upon treatment with high concentration of 2-5A, arising despite the presence of L*. B-C. Biotin-labeled 2-5A was immobilized on streptavidin-coated SPR gold chips. (B) Mouse (mu)RNase L (2.5 μM) or (C) human (hu)RNase L (2.5 μM) were injected in the absence or presence of different concentrations of L* as indicated. D. His_6_-L* binding was immobilized on Ni-NTA chips and mouse RNase L (2.5 μM) was injected in the absence or presence of different concentrations of 2-5A as indicated. RU, response units. E. Kinetic parameters of interactions between mouse RNase L and L* or 2-5A. F-G. Histograms showing mean relative fluorescence units (RFU) resulting from degradation of a FRET RNA probe by RNase L. Experiments were repeated twice in triplicates. F. Mouse RNase L (100 nM) was pre-incubated with mock (circle) or 10μM (triangle) of L* followed by addition of varying concentration of 2-5A p3A3. Graph showing the mean and SEM of RNase L activity and table showing inferred Vmax (maximum velocity), K50 (concentration for half activation) and statistical significance of the differences between the two conditions (unpaired t-test). Ns: non-significant G. Mouse RNase L was either pre-incubated with varying concentration of L* (circle) followed by addition of 3nM of 2-5A or pre-incubated with 3 nM 2-5A followed by addition of varying concentration of L* (triangle). Graph showing mean and SEM of RNase L activity and table showing inferred IC_50_ (half maximal inhibitory concentration) and statistical significance of the difference between the two conditions (unpaired t-test).

Dimerization inhibition could result either from steric hindrance of RNase L protomer association or from inhibition of 2-5A binding to RNase L ankyrin repeats. Our data suggest the latter explanation. Indeed, as shown in Fig 7A (white arrows), high doses of 2-5A added to lysates of 293T cells overexpressing L* and Flag-mouse RNase L could overcome L*-mediated inhibition of RNase L activity. To analyze the influence of L* on 2-5A binding to RNase L in a more direct manner, surface plasmon resonance (SPR) experiments were performed with purified recombinant L*, mouse RNase L and, as a negative control, human RNase L. First, by immobilizing biotin-modified 2-5A on streptavidin (SA)-chips followed by addition of mouse RNase L, we determined that mouse RNase L rapidly binds to 2-5A (*k*_a_ = 2.1 x 10^5^ M^-1^ x sec^-1^) with a relatively slow dissociation rate of 2.2 x10^-3^ sec^-1^ (Fig 7). Direct binding of mouse RNase L or human RNase L to (His)_6_-L* immobilized on Ni-NTA sensor chip was then assessed. Consistent with our prior observations (12), we found that human RNase L did not bind to L* while mouse RNase L bound to L* with a *K*_D_ of 1.3 x 10^−6^ M (S3A and S3B Fig, Fig 7E). Similarly, L* bound to immobilized mouse GST-RNase L (S3C Fig, Fig 7E). Next, to determine if L* binding interfered with 2-5A binding to mouse RNase L, biotin-labeled 2-5A was immobilized on SA-chips and mouse RNase L or human RNase L was simultaneously injected without or with increasing concentrations of L* and sensograms were recorded. Whereas L* efficiently inhibited binding of mouse RNase L to 2-5A (IC_50_ = 6.0 x 10^-7^M), L* did not inhibit human RNase L from binding with 2-5A (Fig 7B and 7C, respectively). Additionally, L* was immobilized and mouse RNase L was injected in the absence or presence of different concentrations of 2-5A. 2-5A dose-dependently inhibited mouse RNase L binding to L* (IC_50_ = 1.32 x 10^−8^ M) (Fig 7D). Taken together, our data show that L* interferes with 2-5A binding to mouse RNase L while no effect of L* was observed on 2-5A binding to human RNase L.

To further characterize the inhibition by L* of 2-5A-dependent RNase L activation, fluorescence energy transfer (FRET) assays based on the degradation of a labeled RNA probe [44] were performed in solution (Fig 7F and 7G). First, purified mouse RNase L was pre-incubated without L* (Fig 7F, open circles) or with 10 μM L* (Fig 7F, closed circles) followed by addition of increasing 2-5A concentrations. Results confirmed that L* inhibits 2-5A-dependent RNase L activation and showed that while the 2-5A concentration for half activation (*K*_50_) remained unchanged, the maximum velocity (*V*_max_) of the RNase L enzymatic activity was decreased by ~40% by L* (Fig 7F). Next, mouse RNase L was either pre-incubated with increasing L* concentrations followed by addition of 3 nM 2-5A (Fig 7G, circles) or pre-incubated with 3 nM 2-5A followed by addition of increasing L* concentrations (Fig 7G, triangles). In both cases L* inhibited 2-5A-dependent RNase L activation but efficacy of the inhibition was more potent when L* was added prior to 2-5A (IC_50_: 9.99 μM vs 34.14 μM). Altogether, these results suggest that L* inhibits 2-5A binding to mouse RNase L, possibly because L* inhibits dimerization required for cooperative binding of 2-5A between the RNase L protomers [30, 45].

### L* can substitute for MHV ns2 anti-RNase L activity in bone marrow-derived macrophages and in the liver of infected mice

**Fig 8:**
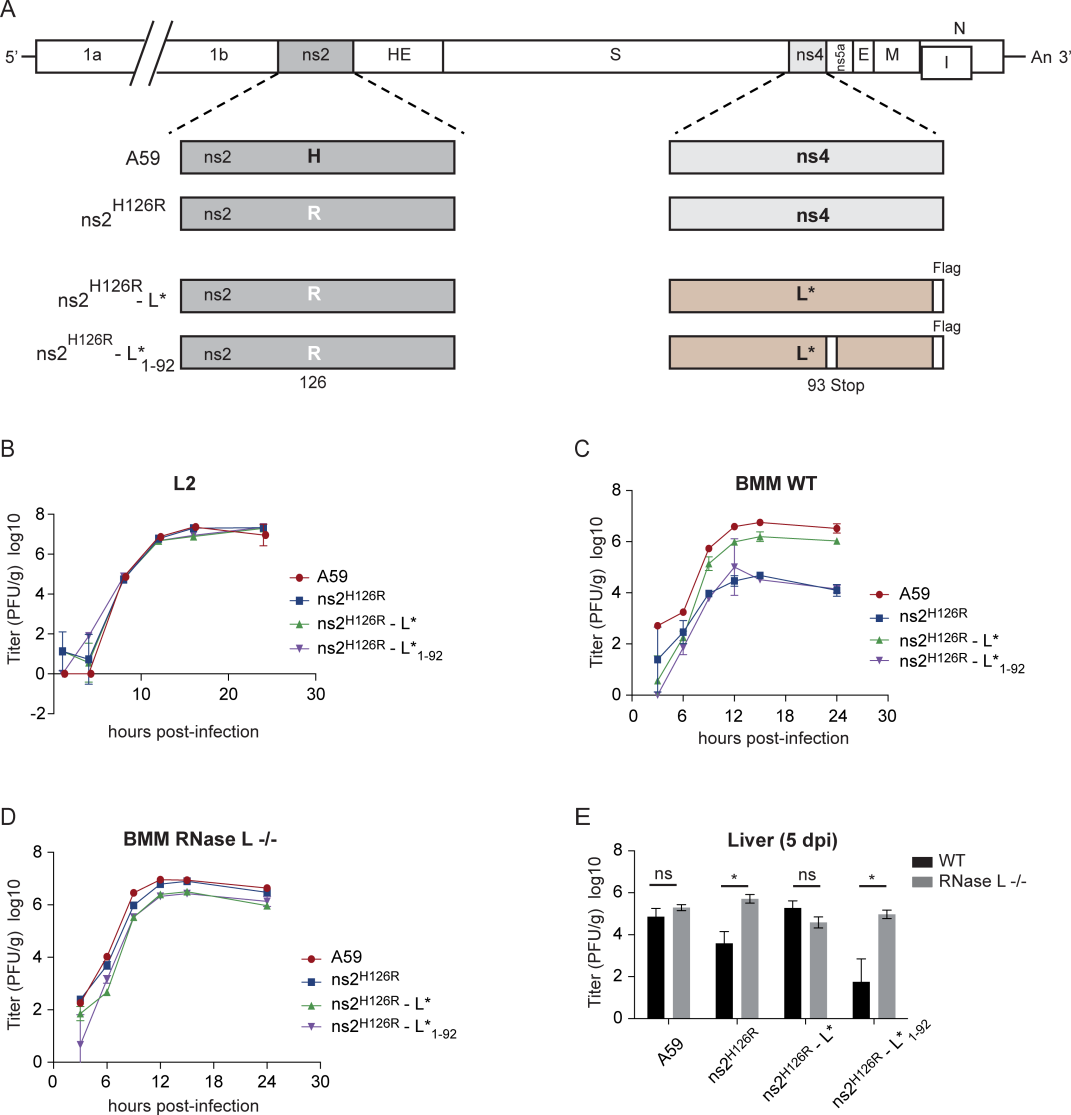
L* compensates ns2 RNase L antagonist activity in bone marrow-derived macrophages (BMM) and in mice. A. Schematic diagram of recombinant MHV. B. L2 fibroblasts were infected (1 PFU/cell). At indicated time points post-infection, virus titers in the cell lysates combined with supernatants were determined by plaque assay (n = 3). C-D. BMM, derived from WT or RNase L−/− mice were infected (1 PFU/cell). At indicated time points post-infection, titers of viruses in the cell lysates combined with supernatants were determined by plaque assay (n = 3). E. Four-week-old WT or RNase L−/− B6 mice were inoculated intrahepatically with WT A59, mutant and chimeric viruses (2000 PFU/mouse). At 5 d.p.i., organs were harvested, homogenized and virus titers determined by plaque assay (n = 4 or 5). Statistics were done using the Mann-Whitney test. Error bars represent standard error of the means.

We next asked whether L* displayed anti-RNase L activity *in vivo*, and in doing so, if L* could substitute for another antagonist of the OAS/RNase L pathway, acting upstream in the OAS/RNase L signaling pathway. L*-mediated inhibition of RNase L *in vivo* was not analyzed for TMEV, by comparing the infection of RNase L-deficient (RNase L^-/-^) and RNase L ^+/+^ mice [21] because these mice are on the C57BL/6 background and therefore rapidly clear TMEV infection through a potent H-2^b^-restricted cytolytic T lymphocyte response [46–48]. We thus tested the RNase L antagonist capacity of L* in chimeric MHV viruses. We constructed recombinant MHV A59 viruses (Fig 8A) carrying either a wild-type or a catalytically defective (H126R mutation) ns2 PDE (an enzyme that degrades 2-5A). From the latter construct, we obtained chimeric viruses where the non-essential NS4 protein was replaced by either wild-type L* (ns2^H126R^-L*) or a truncated L*_1–92_ lacking anti-RNase L activity (ns2^H126R^-L*_1–92_) [12].

The replication ability of these viruses was first assessed in L2 fibroblasts in which IFN-β expression is not induced by MHV [49]. WT, mutant, and chimeric viruses all replicated with comparable kinetics and to similar final titers, indicating that neither the mutations in ns2 nor the replacement of NS4 by L* affected viral replication (Fig 8B).

Virus replication was next evaluated in bone marrow derived macrophages (BMMs), cells where MHV induces the expression of IFN, and in which ns2 PDE activity is required for an efficient replication [33, 49]. We confirmed that the ns2^H126R^-L* chimeric virus expressed L* in these cells (S4A Fig, lane 4) and that L* could inhibit RNase L-mediated RNA degradation (S4B Fig, lane 4). As shown previously, replication of the ns2^H126R^ mutant was severely impaired in BMMs as compared to that of A59 WT virus (Fig 8C). Interestingly, expression of full length L* but not of L*_1–92_ rescued replication of the ns2^H216R^ mutant virus to near WT levels. In contrast, mutant and chimeric viruses replicated efficiently in RNase L^-/-^ BMMs, with similar kinetics and final titers (Fig 8D), confirming that ns2 and L* acted through antagonism of the OAS/RNase L pathway.

Then, WT and RNase L^-/-^ C57BL/6 mice were infected intrahepatically with 2000 PFU of WT A59, mutant and chimeric viruses and viral titers in the liver were measured by plaque assay on mouse L2 cells at 5 days post-infection [50](Fig 8E). As observed in BMMs, ns2 mutant virus (ns2^H126R^) replication in the liver of WT mice was impaired as compared to that of A59 WT virus whereas ns2^H126R^-L* replicated to nearly WT levels. In contrast mutant L*_1–92_ was unable to confer robust replication when expressed from ns2^H126R^-L*_1–92._ All viruses showed the same robustness in the liver of RNase L^-/-^ animals. These results show that the RNase L antagonistic activity of L* can replace the PDE function of ns2 in BMMs and *in vivo*, in the liver of WT C57BL/6 mice.

## Discussion

The OAS/RNase L pathway confers protection against many RNA viruses as well as some DNA viruses and, as a consequence, viruses developed numerous strategies to escape its antiviral effects (for review, see [34, 35]). We recently reported that TMEV L* protein antagonizes RNase L activity through direct protein-protein interaction, suggesting that L* could act by a yet to be described mechanism [12].

A fusion protein carrying ANK R1 and R2 but not either ANK repeat alone readily co-immunoprecipitated with L*, suggesting that these two repeats are both necessary and sufficient to mediate the interaction with L*. On the one hand, replacing two amino acids stretches (26–28 and 47–51) of human RNase L by the corresponding mouse RNase L residues was sufficient to trigger interaction with L* and efficient L*-mediated inhibition of human RNase L. On the other hand, rat/mouse RNase L chimeras showed that residues 80, 81 in ANK R2 were also critical for L* interaction with RNase L and, to a lesser extent, for RNase L inhibition. In general, results from interaction and functional studies were congruent. For some constructs, however, interaction was observed while inhibition was weak, suggesting that a strong affinity is required for RNase L inhibition. In summary, our data show that L* inhibits RNase L activation by associating with loops exposed at the surface of RNase L ANK R1 and R2 (Fig 4). The fact that these loops diverge among RNase L from different species is explaining the exquisite species specificity of L* activity.

Using crosslinking experiments, we showed that L* prevented RNase L dimer and oligomer formation. Oligomerization of human RNase L was suggested by Han et al. [29] but was however not observed in the case of wild-boar RNase L [30]. In our experiments, oligomerization was observed after overexpression of RNase L in 293T cells (Fig 6). Yet, since the process depended on 2-5A, oligomer formation did not merely result from RNase L overexpression and were likely formed by association of dimers. It is not known whether 2-5A mediates the interface between RNase L dimers and/or whether dimers interact with each other through a novel interface. Mass spectrometry data suggest, however, that no additional protein is included in the RNase L oligomers.

Surface plasmon resonance experiments revealed that L* interferes with 2-5A binding to RNase L. Mouse RNase L bound to L* with a *K*_D_ of 1.3 x 10^−6^ M. On one hand, increasing L* concentrations decreased mouse RNase L binding to immobilized 2-5A (Fig 7B). On the other hand, increasing 2-5A concentrations decreased mouse RNase L binding to immobilized L* (Fig 7D), which is compatible with a competition between L* and 2-5A for RNase L binding.

In solution FRET assays for RNase L activity, however, do not support a competitive binding model. Data confirmed that L* counteracted RNase L activation by 2-5A but the *V*_max_ of the RNase L enzymatic activity was significantly decreased while the 2-5A concentration for half activation (*K*_50_: ~2nM) was preserved (Fig 7F). Also, L* inhibited 2-5A-dependent RNase L activation when it was added both prior or after 2-5A addition but the inhibition was stronger when L* was added first (Fig 7G). Taken together, our data are compatible with a model where L* binding to RNase L sterically hinders 2-5A binding to ANK repeats of RNase L. However, pre-bound 2-5A would decrease the affinity of L* for RNase L, likely through dimerization of RNase L. If our model is correct, RNase L activation/inhibition would depend on a race between 2-5A and L* production in infected cells. It is worth noting that L* is produced by an open reading frame (ORF) that overlaps the ORF coding the main viral polyprotein. The L* ORF can be translated from the viral genomic RNA in an IRES-dependent fashion, and is likely expressed very early after infection, before viral dsRNA produced during RNA replication can trigger OAS activation.

It is intriguing to observe the variety of strategies developed by viruses to antagonize RNase L. The NS1 protein of influenza A virus sequesters dsRNA thus preventing sensing by OAS [36]. DsRNA sequestration allows the virus to use a single activity to interfere with OAS activation and to prevent activation of other dsRNA-dependent pathways such as the RIG-like helicase pathway that leads to IFN production and that of the double-stranded RNA-dependent *protein* kinase (PKR) [51], which leads to translation inhibition. Conversely, acting downstream of the OAS/RNase L pathway would represent a more specific manner to efficiently antagonize RNase L. It is expected that such a strategy would be used by viruses that are particularly susceptible to the OAS/RNase L pathway. In this respect, it is puzzling that L* evolved to bind RNase L, and yet interferes with 2-5A binding rather than preventing RNase L dimerization directly. Indeed, the unique known function of 2-5A is RNase L activation [52]. Thus, inhibiting dimerization would appear as a more likely mechanism to antagonize RNase L activation because the concentration of RNase L monomers is expected to be much lower than that of 2-5A molecules, which can reach micromolar concentrations in infected cells [53, 54].

MHV, Rotaviruses and MERS-CoV also inhibit the OAS/RNase L pathway at the level of 2-5A by producing viral PDEs that degrade 2-5A [33, 37, 38]. A hypothesis is that complete RNase L inhibition would be detrimental for these viruses so that they evolved in a way to inhibit RNase L in a regulated manner, according to the concentration of the antagonist protein. For instance, products generated by RNase L enzymatic activity can promote autophagy. Induction of autophagy by RNase L was reported to dampen EMCV or SeV replication at early stages of infection whereas it promoted viral replication at later stages [24, 25], possibly by increasing the supply in autophagosomal membranes that are hijacked by some viruses like EMCV [55] to assemble their replication complexes.

Using chimeric MHV expressing L*, we showed that L* could rescue the replication of MHV mutants lacking PDE activity, both in primary macrophages and in the liver of infected mice.

On the one hand, this result shows that L* readily antagonizes RNase L activity *in vivo*, in infected mouse tissues. On the other hand, it suggests that proteins that act on distinct steps of the OAS/RNase L pathway can substitute for each other. ns2 triggers 2-5A degradation while L* prevents their association with RNase L. The fact that L* can substitute for ns2 therefore supports the idea that 2-5A does not play critical functions affecting viral replication other than activating RNase L.

In conclusion, we identified a novel evasion mechanism of the antiviral OAS/RNase L pathway, which surprisingly involves interference of 2-5A binding to RNase L by a viral protein.

## Materials and methods

### Ethics statement

This study was carried out in strict accordance as defined in the federal regulations set forth in the Animal Welfare Act (AWA), the recommendations in the Guide for the Care and Use of Laboratory Animals of the National Institutes of Health, and the guidelines of the University of Pennsylvania Institutional Animal Use and Care Committee. The protocols were approved (# 803711) by the Institutional Animal Care and Use Committee at the University of Pennsylvania.

### Cells

HeLa-M [41], and 293T (DuBrigde, 1987) cells were cultured in Dulbecco modified Eagle medium (Lonza, Cat N°: BE12-604F) containing 4.5g/L glucose and L-glutamine and supplemented with 10% fetal calf serum. BHK-21 (ATCC) cells were cultured in Glasgow-minimal essential medium (Gibco, ThermoFisher Scientific, Cat N°: 11710035) supplemented with 10% newborn calf serum and 2.95g/L tryptose phosphate broth. Both media were supplemented with 100U/mL penicillin and 10μg/mL streptomycin. Primary bone marrow-derived macrophages (BMM) were generated from the hind limbs of B6 or RNase L^–/–^mice as described previously [33] and cultured in DMEM supplemented with 10% FBS and 20% L929 cell-conditioned medium for 6 days before infection. Cultures were routinely ≥99% pure as assessed by positive staining for expression of CD11b and negative staining for expression of CD11c.

### Vectors and viruses

Expression vectors used in this study are summarized in Table 1.

**Table 1:** Expression plasmids used in this study^1^.

L* expressing vectors			
**Name**	**Tag**	**Encoded protein, particularity**	**Type (parental vector)**
pFS105	N-term His	L*_DA_ WT	Plasmid (pET-15b)
pMD01	/	L*_DA_ WT (-IRES-mCherry)	Lentiviral (pTM945)
pMD06	/	L*_RTV-1_ WT (-IRES-mCherry)	Lentiviral (pTM945)
pMD11	N-term HA	L*_DA_ WT	Lentiviral (pTM898)
pMD12	N-term HA	L*_DA_ stop codon 93	Lentiviral (pTM898)
pMD15	N-term HA	L*_RTV-1_ WT	Lentiviral (pTM898)
**Mouse RNase L expressing vectors**			
**Name**	**Tag**	**Encoded protein, particularity**	**Type (parental vector)**
pFS165	N-term Flag	MuRNase L WT	Plasmid (pcDNA3)
pFS165B	N-term cleavable GST	MuRNase L WT	Plasmid (pGEX4T1)
pFS178	/	MuRNase L WT	Plasmid (pDEST15)
pMD25	N-term Flag	MuRNase L—human 1–157	Plasmid (pcDNA3)
pMD33	N-term Flag	MuRNase L—human 1–86	Plasmid (pcDNA3)
pMD39	N-term Flag	MuRNase L—human 1–58	Plasmid (pcDNA3)
pMD50	C-term Flag	MuRNase L WT	Plasmid (pcDNA3)
pMD57	N-term Flag	MuRNase L–G47, N50, V51	Plasmid (pcDNA3)
pMD63	N-term Flag	MuRNase L—human 26–51	Plasmid (pcDNA3)
pMD65	N-term Flag	MuRNase L–N26, H27, L58	Plasmid (pcDNA3)
pMD67	N-term Flag	MuRNase L—N26, H27, L58, G47, N50, V51	Plasmid (pcDNA3)
pMD69	C-term Flag	MuRNase L—K391R	Plasmid (pcDNA3)
pMD70	N-term Flag	MuRNase L—rat 18–58	Plasmid (pcDNA3)
pMD78	N-term Flag	MuRNase L—rat 1–84	Plasmid (pcDNA3)
pMD82	N-term Flag	MuRNase L—R80, Y81	Plasmid (pcDNA3)
pMD87	N-term Flag	MuRNase L—Y27, L28, Q47, R80, Y81	Plasmid (pcDNA3)
pMD94	N-term Flag	MuRNase L W80A	Plasmid (pcDNA3)
pMD95	N-term Flag	MuRNase L K89A	Plasmid (pcDNA3)
pMD96	N-term Flag	MuRNase L F126A	Plasmid (pcDNA3)
pMD97	N-term Flag	MuRNase L E131A	Plasmid (pcDNA3)
pMD98	N-term Flag	MuRNase L R155A	Plasmid (pcDNA3)
pMD99	N-term Flag	MuRNase L R309A	Plasmid (pcDNA3)
pMD100	N-term Flag	MuRNase L H311A	Plasmid (pcDNA3)
**Human RNase L expressing vectors**			
**Name**	**Tag**	**Encoded protein, particularity**	**Type (parental vector)**
pFS183	N-term Flag	HuRNase L WT	Plasmid (pcDNA3)
pMD26	N-term Flag	HuRNase L—mouse 1–157	Plasmid (pcDNA3)
pMD34	N-term Flag	HuRNase L—mouse 1–86	Plasmid (pcDNA3)
pMD40	N-term Flag	HuRNase L—mouse 1–58	Plasmid (pcDNA3)
pMD51	C-term Flag	HuRNase L WT	Plasmid (pcDNA3)
pMD58	N-term Flag	HuRNase L—K47, D50, A51	Plasmid (pcDNA3)
pMD64	N-term Flag	HuRNase L—mouse 26–51	Plasmid (pcDNA3)
pMD66	N-term Flag	HuRNase L—D26, S27, S28	Plasmid (pcDNA3)
pMD68	N-term Flag	HuRNase L—D26, S27, S28, K47, D50, A51	Plasmid (pcDNA3)
**Rat RNase L expressing vectors**			
**Name**	**Tag**	**Encoded protein, particularity**	**Type (parental vector)**
pMD27	N-term Flag	RatRNase L WT	Plasmid (pcDNA3)
pMD71	N-term Flag	RatRNase L—mouse 18–58	Plasmid (pcDNA3)
pMD79	N-term Flag	RatRNase L—mouse 1–84	Plasmid (pcDNA3)
pMD83	N-term Flag	RatRNase L—S80, H81	Plasmid (pcDNA3)
pMD88	N-term Flag	RatRNase L—S27, S28, K47, S80, H81	Plasmid (pcDNA3)
**Others**			
**Name**		**Encoded protein, particularity**	**Type (parental vector)**
pMD41	N-term Flag	MuANK R1-eGFP	Plasmid (pcDNA3)
pMD74	N-term Flag	MuANK R1-4-eGFP	Plasmid (pcDNA3)
pMD80	N-term Flag	MuANK R1-2-eGFP	Plasmid (pcDNA3)
pMD84	N-term Flag	MuANK R1-2-eGFP	Lentiviral (pTM898)
pMD85	N-term Flag	HuANK R1-2-eGFP	Plasmid (pcDNA3)
pMD86	N-term Flag	RatANK R1-2-eGFP	Plasmid (pcDNA3)
pMD93	N-term Flag	MuANK R2-eGFP	Plasmid (pcDNA3)
pTM898	/	MCS—IRES—Neo	Lentiviral
pTM945	/	MCS—IRES—mCherry	Lentiviral

^1^All constructs allowed expression of the Neo (G418/Geneticin) resistance gene

MCS: multiple cloning site, IRES: internal ribosome entry site, N-term: N-terminal, C-term: C-terminal, Mu: murine, Hu: human.

TMEV derivatives were produced by electroporation of BHK-21 cells with the genomic RNA transcribed *in vitro* from plasmids carrying the corresponding full-length viral cDNA.

Viruses were derived from the DA1 molecular clone (Genbank accession JX443418). A recombinant TMEV cDNA, named pSV28, was constructed, carrying the extra sequence coding for the HA epitope at the N-terminus of L* GGT ACC CGT ACG ACG TTC CGG ACT ACG CGC TGC TTG TAA GCA CGG between nucleotides 1081 and 1082 of pVV18 (pTMDA1 derivative, macrophage-adapted). DA1 mutant expressing a truncated L* (L*_1–92_), named pSV31 was obtained by introduction of a stop codon mutation in pSV28. This mutation prevents the expression of full-length L* but does not affect the translation of the main viral open reading frame encoding the polyprotein. The corresponding viruses SV28 and SV31 were produced by reverse genetics and titrated by plaque assay. HeLa-M cells were typically infected at a MOI of 5 PFU per cell for 16 hours.

The lentiviral vector pMD84 (Table 1) was obtained by introduction of the sequence coding for Flag-ANK1-2-eGFP from pMD80 into pTM898 [12], a derivative of pCCL.sin.cPPT.hPGK.GFP.pre [56]. Viral particles were produced as described [57], pseudotyped with the glycoprotein of vesicular stomatitis virus (VSV-G). Transduced HeLa-M cells were selected with G418 (1mg/mL) and transgene expression was verified by controlling the eGFP green fluorescence using fluorescence microscopy.

Chimeric MHV-L* viruses. A series of chimeric and recombinant viruses were constructed using the full-length infectious cDNA clone of MHV strain A59 as previously described [33, 58]. These viruses included A59 WT, and the A59 recombinant virus expressing a functional ns2 mutation in which the catalytic His residue at position 126 is mutated to Arg(ns2^H126R^). Additionally, two chimeric A59- ns2^H126R^ -L* viruses were also assembled where L* is inserted in place of the nonessential NS4 protein. These viruses also expressed ns2^H126R^ and either L*WT or another L* protein in which a stop codon (UAA) had been inserted at amino acid position 93 that renders it inactive (L*_1–92_) [8, 10]. A sequence coding a Flag epitope tag was inserted at the 3’ end of the L* coding region_._

### Transfection

Plasmid DNA were transfected using TransIT LT-1 transfection reagent according to the manufacturer’s protocol (Mirus, Cat N°: 11668019).

Cells plated in 24 well-plates were transfected with 0.3 μg/mL polyI:C (Amersham-Pharmacia) with 2 μL Lipofectamine 2000 transfection reagent according to the manufacturer’s protocol (Invitrogen, 11668019).

### Coimmunoprecipitation assays

Flag-RNase L and N-terminally HA-tagged L* were coexpressed in 293T cells from transfected expression plasmids (Table 1). Coimmunoprecipitation assays were conducted on 293T cell lysates, as previously described [12] except that protein A/G Magnetic Beads (Pierce, 88803) were used instead of A/G Ultralink Resin. HA-L* proteins were immunoprecipitated with anti-HA antibody (Covance, MMS101-P) and immunoprecipitated proteins were detected using SDS-PAGE and immunoblot analysis using anti-HA and anti-Flag (Sigma-Aldrich M8823) antibodies. As a control, total cell lysate corresponding to 10% of the input used for immunoprecipitation was also analyzed by immunoblotting using anti-HA, anti-Flag and anti-GAPDH (Millipore, MAB374) antibodies. Bands corresponding to coimmunoprecipitated Flag-RNase L were quantified from scanned immunoblot using image J or from CCD images using Bio-1D software (Vilber).

### Immunofluorescence

HeLa-M cells were grown on poly-L-lysine coated coverslips before transfection or infection. Twenty-four hours post-transfection or 16 hours post-infection, cells were fixed for 5 min with paraformaldehyde 4% in PBS then permeabilized for 5 min with Titron X-100 0.1% in PBS and unspecific antigens were blocked for 1h using 2% normal goat serum (Sigma-Aldrich S2007) in PBS. Cells were then incubated for 1 hour with primary antibodies at a dilution of 1:50 in the same buffer (anti-L*, adsorbed rabbit polyclonal) or 1:25 (anti-VP1-F12B3, mouse monoclonal). Cells were intensively washed with Tween 20 0.01% in PBS and incubated for 1 hour with species-matched AlexaFluor-conjugated secondary antibodies (Invitrogen A-31573, A-11036) at a dilution of 1:800 in the same buffer.

For mitochondrial staining, cells were incubated for 45 min before fixation with 200 nM MitoTracker Red CMXRos (Molecular Probes, M7512). Coverslips were mounted on slides with Mowiol 4–88 medium (Calbiochem, 475904) and analyzed by fluorescence microscopy using a spinning disk confocal microscope equipped with an Axiocam MRm camera (Zeiss). Intensity, contrast, and color balance of images were equilibrated using Zen (Zeiss) and Adobe Photoshop.

### RNA degradation assay

Flag-RNase L and L* constructs (Table 1) were coexpressed in HeLa M or 293T cells. OAS was activated by polyI:C transfection (which produces 2-5A that activates RNase L) for 7 hours. RNA was isolated according to the method of Chomczynski & Sacchi [59]. RNA degradation was assessed by agarose gel electrophoresis in Tris-sodium acetate-EDTA (TAE) buffer or by running RNA samples on RNA nano 6000 microfluidics chips run on a 2100 bioanalyzer (Agilent Technologies). Bands corresponding to RNA degradation products were quantified from gel-like images using image J.

### Dimerization assay

To assess RNase L dimerization, RNase L-Flag expression vectors were transfected in 293T cells along with L* expression plasmids or equivalent empty vectors. C-terminally flagged RNase L were used because we noticed that detection of N-terminally flagged constructs by immunobloting was less consistent in this assay. Twenty four hours post-transfection, cells were washed with phosphate-buffered saline (PBS) and harvested in HEPES lysis buffer (HEPES 20mM pH7.5, NaCl 100mM, NP40 0.5%, EDTA 2mM, PMSF 1mM). Cells were disrupted by 10 passages through a 21G needle and lysates were centrifuged at 12,000x g for 5min at room temperature. 2-5A was then added in the supernatant at different final concentrations (from 0.25 to 8 μM ATP equivalent 2-5A). After 10 min at 37°C, crosslinking of interacting proteins was performed by addition of 5mM disuccinimidyl suberate (DSS, ThermoScientific, 21658) for 30 min. Finally the crosslinking reaction was quenched with 50mM Tris-HCl pH7.5, RNA was extracted from 1/6^th^ volume of the lysate as a control for RNase L activation and protein samples were prepared by addition of Laemmli buffer to the remaining lysate. RNase L activation was controlled by rRNA degradation analysis on RNA gel electrophoresis and RNase L dimerization was analyzed by immunoblotting using an anti-Flag antibody (Sigma-Aldrich M8823).

For analysis of the oligomeric bands by mass spectrometry, the experiment was repeated using 2mM dithiobis(sulfosuccinimidyl propionate) (DTSSP, CovaChem, 13304), a cleavable protein crosslinking reagent. RNase L-Flag oligomers were then immunoprecipitated using an anti-Flag antibody (Sigma-Aldrich M8823) and protein samples were prepared by addition of a non-reducing protein buffer (Laemmli buffer without 2-mercaptoethanol).

### FRET assays for RNase L activity

RNase L activity was determined using fluorescence resonance energy transfer (FRET) assays as described previously [12, 44]. In Fig 7F, the recombinant murine RNase L (100 nM) was pre-incubated with purified L* (10 μM) for 30 min on ice followed by addition of increasing concentration of p3(2–5)A3. In Fig 7G, mouse RNase L was pre-incubated with varying concentration of L* followed by addition of 3nM of p3(2–5)A3. Alternatively, RNase L was pre-incubated with 3 nM p3(2–5)A3 followed by addition of varying concentration of L*. RNase L activity was monitored with cleavage of dual labeled probe [12, 44]. Best-fit curves and associated kinetic parameters were established using a nonlinear regression model with GraphPad Prism software.

### Mass spectrometry

After co-immunoprecipitation, samples were resolved using an 8% Tris-Glycine SDS-PAGE and proteins were visualized using Coomassie Blue (PageBlue™ Protein Staining Solution, Thermo Scientific, 24620). Bands of interest were cut out from the gel, reduced with DTT, alkylated with chloroacetamide and digested with chymotrypsin. The peptides were analyzed, as previously described [60], by capillary LC-tandem mass spectrometry in a LTQ XL ion trap mass spectrometer (ThermoScientific, San Jose, CA) fitted with a microelectrospray probe. The data were analyzed with the ProteomeDiscoverer software (ThermoScientific, version 1.4.1), and the proteins were identified with SequestHT against a target-decoy nonredundant human and mouse proteins database obtained from Uniprot. The false discovery rate was set below 5% and the number of unique peptides was set higher or equal to 2.

### L* and RNase L recombinant protein expression and purification

The plasmid L*-pET15b was expressed in *E*. *coli* TB1 (NEB, Inc.). Bacteria were grown in LB containing 100 μg/ml ampicillin at 37°C, and shaked at 250 rpm until the OD_600nm_ reached 0.7. The cultures were cooled on ice, induced using 0.5 mM IPTG and further grown for 6 h at 20°C. Cells were harvested and washed with ice cold 20 mM HEPES pH 8.2 containing 150 mM NaCl and lysed by sonication (20 x 10s) in buffer A (20 mM HEPES pH 8.2, 10% glycerol, 2 mM EDTA, 14.2 mM beta-mercaptoethanol, and 200 mM NaCl) containing 10 units/ml benzonase, protease inhibitor cocktail (Roche) and 100μM PMSF. Lysates were clarified by centrifugation at 10,000 x g for 30 min at 4°C, diluted 5-fold and bound to SP Sepharose FF cation exchange resin (GE Lifesciences) packed in XK 15mm x 10mm column. The column was washed with binding buffer A until the basal OD_280nm_ was constant. The bound proteins were eluted with a 0.2–2 M gradient of NaCl in buffer A. The fractions were analyzed by SDS page (L* eluted at >500 mM NaCl). The peak fractions containing L* were pooled and diluted 1:3 in buffer A and bound with Ni-NTA resin, washed with buffer A containing 10 mM imidazole and eluted with 150 mM imidazole in buffer A. Fractions were loaded on 12% SDS-PAGE and stained with Gel-Code Blue (Promega). The yield was ~0.25mg/L.

Human RNase L was produced as described [61]. Murine RNase L was cloned as an N-terminal GST fusion in the pGEX4T1 vector (GE lifesciences). The recombinant protein was produced in *E*. *coli*, as described [27] and the GST tag was proteolytically removed if required.

### Synthesis, purification and biotin labeling of 5′-triphosphoryl, 2′–5′-linked oligoadenylates (2–5A)

2–5A (p_3_(A2′p)_n_A, where n = 1 to ≥3) was enzymatically synthesized from ATP using hexahistidine-tagged and purified recombinant porcine 42-kDa 2-5A synthetase (OAS1). Individual 2-5A oligomers were purified (>95% purity) using a Dionex PA100 (22 mm × 250 mm) semi-preparative column interfaced with a Beckman system gold HPLC system under the control of 32-Karat workstation [62]. The 5’-triphosphorylated triadenylate, p_3_A2’p5’A2’p5’A, was used for RNase L activation. Biotinlyation of 2-5A was performed using periodate chemistry as described earlier [30, 44].

### Surface plasmon resonance (SPR)

Kinetic characterization of L* binding to RNase L and inhibition of 2-5A binding to RNase L were monitored by SPR with a Biacore 3000 (GE Healthcare). Response units (RU), a measure of binding, were monitored as a function of time.

In some experiments, biotin labeled 2-5A immobilized on streptavidin (SA)-chips was used with mouse RNase L or human RNase L in the absence or presence of different concentrations of L*. L* and 2-5A did not interact with each other as the signal was not influenced by L* in Fig 7C. In other experiments, (His)_6_-L* protein was immobilized on Ni-NTA chips, according to the manufacturer’s protocol (GE Health Care) and used for direct binding with human RNase L, mouse RNase L, or with mouse RNase L (2.5 μM) in the presence of different concentrations of 2-5A.

Alternatively, purified anti-GST monoclonal antibody clone P1A12 (BioLegend Inc., San Diego) was immobilized on sensor CM5 chips (GE Healthcare) to achieve a surface equivalent to ~1000 RU. The purified recombinant GST-tagged murine RNase L (2.5 μM) supplemented with 0.005% surfactant P20 (HBS-P SPR buffer) was immobilized on anti-GST antibody coated sensor chips at a flow rate of 10 μl/min for 3 min at 25°C to achieve a surface equivalent to 1000 RU. An additional wash for 5 min at a flow rate of 20 μl/min was performed with buffer alone. L* protein (>90%, SDS-PAGE) was used at 0.1 to 10 μM in SPR buffer HBS-P (GE Healthcare).

In all SPR experiments, analyte solutions of different concentrations were passed over the sensor chip with immobilized protein or 2-5A at a flow rate of 10 μl/min for 3 min, and association was monitored. Dissociation was monitored using SPR buffer for an additional 5 min. Data was normalized against a reference channel immobilized with GST alone. Analysis and fitting was performed with BIAEvaluation software, version 3.2 (Biacore Inc.), with the option for simultaneous *k*_*a*_/*k*_*d*_ calculations. Fitting of sensorgram data was carried out according to global fitting, and the *k*_*a*_ and *k*_*d*_ values were calculated with a 1:1 Langmuir model.

### MHV infections

Infection of BMM. Virus was added to cells at an MOI of 1 PFU/cell and allowed to adsorb for one hour at 37°C. Cultures were washed with PBS (3 times) and then fed with medium as described for each cell type. The culture supernatants were harvested at the times indicated for the specific experiments and titered by plaque assay on L2 cells [33].

Infections of mice *in vivo*. Four- to five-week-old B6 mice or RNase L^–/–^mice were anesthetized with isoflurane (IsoFlo, Abbott Laboratories) and inoculated intrahepatically with 2000 plaque forming units (PFU) of WT A59, ns2^H126R^ or chimeric MHV-L* viruses in 20 μl of PBS containing 0.75% BSA. Organs were harvested at day 5 post infection and viral titers were determined by plaque assay on L2 cells [33].

### Statistical analysis

Statistics were done using the tests indicated in the figure legends using GraphPad Prism. Error bars represent standard error of the means. * indicates a statistically significant difference between indicated conditions (p< 0.05).

## Supporting information

S1 Fig Mouse RNase L interacts with cytosolic but not mitochondrial L*. HeLa-M cells were transduced to stably express Flag-muANK R1-2-eGFP. Transduced cells exhibited a diffuse cytoplasmic and nuclear green fluorescence. L* was then introduced in the cells either by transfection or by infection to assess whether L* expression would trigger the relocation of Flag-muANK R1-2-eGFP to the cytosol and/or the mitochondrial surface. The truncated inactive L*_1–92_ was used as a negative control. A. Design of the experiment. B. Visualization of Flag-muANK R1-2-eGFP fluorescence (green). L*_1–92_ (ctrl-, negative control) expression did not induce any relocation of the fusion protein. L* WT expression led to Flag-muANK R1-2-eGFP relocation to the cytoplasmic compartment. C. L* did not concentrate the fusion protein at the mitochondrial surface. Upper panels: visualization of Flag-muANK R1-2-eGFP fluorescence (green, left), HA-L* (anti-L* serum, artificially colored in blue, middle) and mitochondria (MitoTracker, red, right). Lower panels: merge of Flag-muANK R1-2-eGFP (green) and mitochondria (MitoTracker, red) (left), merge of HA-L* (blue) and mitochondria (MitoTracker, red) (middle), and merge of Flag-muANK R1-2-eGFP (green), L* (blue) and mitochondria (MitoTracker, red) (right).
(PDF)

S2 Fig Inhibition of chimeric RNase L by L*. Analysis of RNase L-mediated RNA degradation in HeLa-M cells overexpressing indicated Flag-RNase L chimera and HA-L*. RNA samples extracted 7 hours after polyI:C transfection were analyzed by RNA chips and quantified. A. Inhibition of mouse RNase L carrying indicated human RNAse L residues (left) and of human RNase L carrying indicated mouse RNAse L residues (right) by L*_DA_. Values under the lanes indicate the extent of rRNA degradation inhibition compared to that of WT mouse RNase L. B. Inhibition of mouse RNase L carrying indicated rat RNAse L residues (left) and of rat RNase L carrying indicated mouse RNAse L residues (right) by L*_DA_ and L*_RTV-1_. Values under the lanes indicate the extent of rRNA degradation inhibition by L* compared to that mediated by L*_DA_ on mouse RNase L (left panel) or compared to that mediated by L*_RTV-1_ on WT rat RNase L (right panel).
(PDF)

S3 Fig Mouse RNase L but not human RNase L binds to L*. Surface plasmon resonance (SPR) experiments were performed using Biacore technology with purified recombinant L*, mouse RNase L and human RNase L. A. His_6_-L* was immobilized on Ni-NTA chips and human RNase L was injected at the indicated concentrations. B. His_6_-L* was immobilized on Ni-NTA chips and mouse RNase L was injected at the indicated concentrations. C. GST-mouse RNase L was immobilized on anti-GST CM5 sensor chip and L* was injected at the indicated concentrations. RU: response units.
(PDF)

S4 Fig Full-length L* was expressed in ns2^H126R^-L*-infected BMMs and inhibited RNase L-mediated RNA degradation. A. Flag-L* expression in infected BMM was detected by immunoblotting using an anti-Flag antibody. B. Analysis of RNase L-mediated RNA degradation was performed by RNA chip.
(PDF)
