# Supplementary material for: A novel mechanism of RNase L inhibition: Theiler's virus L* protein prevents 2-5A from binding to RNase L
Source: PLoS Pathog. 2018 Apr 13;14(4):e1006989. doi: 10.1371/journal.ppat.1006989 (PMC5927464; doi:10.1371/journal.ppat.1006989)
Supplement: S1 Fig — HeLa-M cells were transduced to stably express Flag-muANK R1-2-eGFP. Transduced cells exhibited a diffuse cytoplasmic and nuclear green fluorescence. L* was then introduced in the cells either by transfection or by infection to assess whether L* expression would trigger the relocation of Flag-muANK R1-2-eGFP to the cytosol and/or the mitochondrial surface. The truncated inactive L*1–92 was used as a negative control. A. Design of the experiment. B. Visualization of Flag-muANK R1-2-eGFP fluorescence (green). L*1–92 (ctrl-, negative control) expression did not induce any relocation of the fusion protein. L* WT expression led to Flag-muANK R1-2-eGFP relocation to the cytoplasmic compartment. C. L* did not concentrate the fusion protein at the mitochondrial surface. Upper panels: visualization of Flag-muANK R1-2-eGFP fluorescence (green, left), HA-L* (anti-L* serum, artificially colored in blue, middle) and mitochondria (MitoTracker, red, right). Lower panels: merge of Flag-muANK R1-2-eGFP (green) and mitochondria (MitoTracker, red) (left), merge of HA-L* (blue) and mitochondria (MitoTracker, red) (middle), and merge of Flag-muANK R1-2-eGFP (green), L* (blue) and mitochondria (MitoTracker, red) (right). (PDF) [file ppat.1006989.s001.pdf]

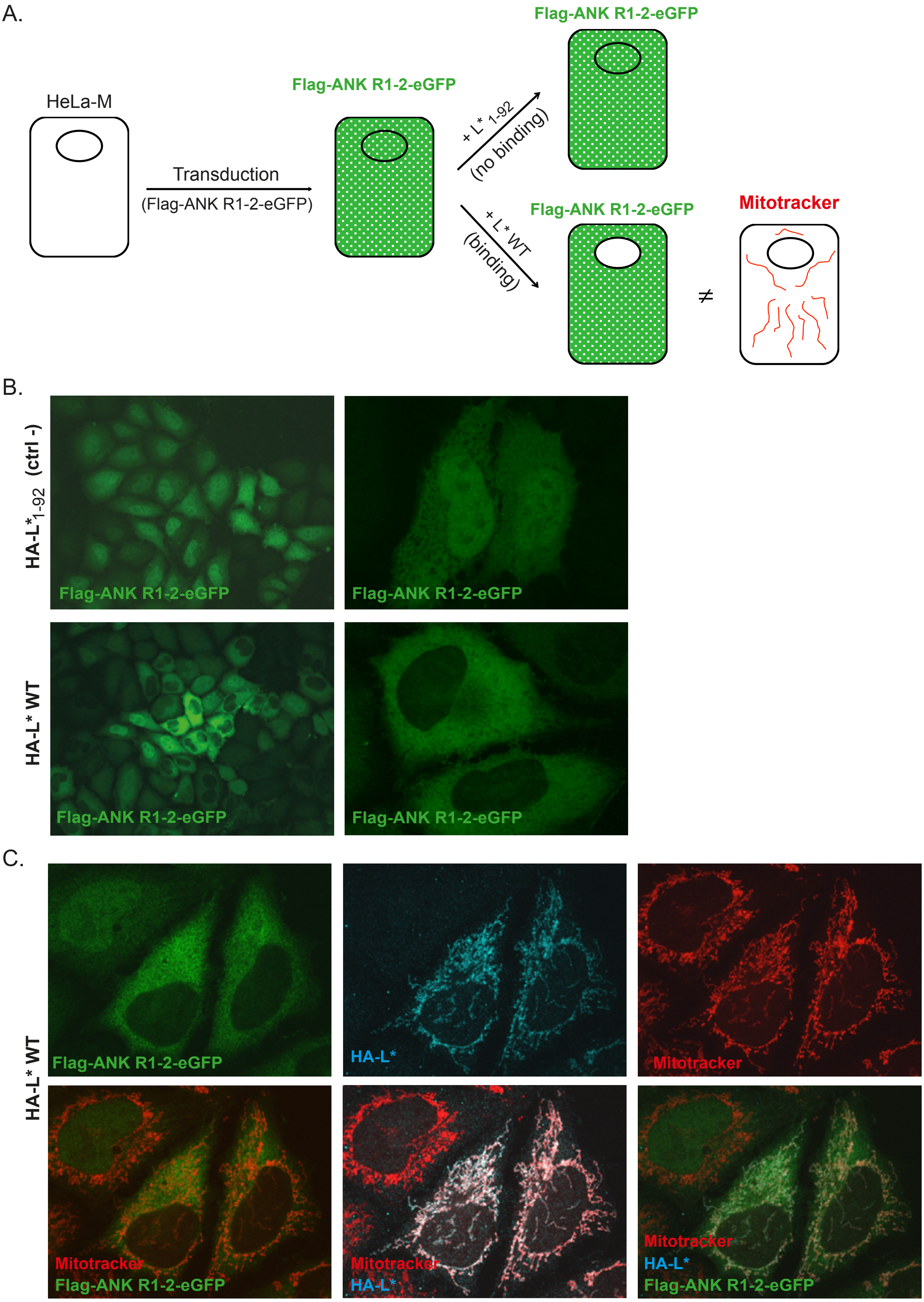

A.
HeLa-M
Transduction
(Flag-ANK R1-2-eGFP)
Flag-ANK R1-2-eGFP
+ L* 1-92
(no binding)
+ L* WT
(binding)
Flag-ANK R1-2-eGFP
Flag-ANK R1-2-eGFP
≠
Mitotracker
B.
HA-L*1-92 (ctrl -)
HA-L* WT
Flag-ANK R1-2-eGFP
C.
HA-L* WT
Flag-ANK R1-2-eGFP
HA-L*
Mitotracker
Mitotracker
Flag-ANK R1-2-eGFP
Mitotracker
HA-L*
Mitotracker
HA-L*
Flag-ANK R1-2-eGFP

S1 Fig

S1 Fig. Mouse RNase L interacts with cytosolic but not mitochondrial L*

HeLa-M cells were transduced to stably express Flag-muANK R1-2-eGFP. Transduced cells exhibited a diffuse cytoplasmic and nuclear green fluorescence. L* was then introduced in the cells either by transfection or by infection to assess whether L* expression would trigger the relocation of Flag-muANK R1-2-eGFP to the cytosol and/or the mitochondrial surface. The truncated inactive L^*_{1-92} was used as a negative control.

A. Design of the experiment.

B. Visualization of Flag-muANK R1-2-eGFP fluorescence (green). L^*_{1-92} (ctrl-, negative control) expression did not induce any relocation of the fusion protein. L* WT expression led to Flag-muANK R1-2-eGFP relocation to the cytoplasmic compartment.

C. L* did not concentrate the fusion protein at the mitochondrial surface. Upper panels: visualization of Flag-muANK R1-2-eGFP fluorescence (green, left), HA-L* (anti-L* serum, artificially colored in blue, middle) and mitochondria (MitoTracker, red, right). Lower panels: merge of Flag-muANK R1-2-eGFP (green) and mitochondria (MitoTracker, red) (left), merge of HA-L* (blue) and mitochondria (MitoTracker, red) (middle), and merge of Flag-muANK R1-2-eGFP (green), L* (blue) and mitochondria (MitoTracker, red) (right).
