# Supplementary material for: A novel mechanism of RNase L inhibition: Theiler's virus L* protein prevents 2-5A from binding to RNase L
Source: PLoS Pathog. 2018 Apr 13;14(4):e1006989. doi: 10.1371/journal.ppat.1006989 (PMC5927464; doi:10.1371/journal.ppat.1006989)
Supplement: S2 Fig — Analysis of RNase L-mediated RNA degradation in HeLa-M cells overexpressing indicated Flag-RNase L chimera and HA-L*. RNA samples extracted 7 hours after polyI:C transfection were analyzed by RNA chips and quantified. A. Inhibition of mouse RNase L carrying indicated human RNAse L residues (left) and of human RNase L carrying indicated mouse RNAse L residues (right) by L*DA. Values under the lanes indicate the extent of rRNA degradation inhibition compared to that of WT mouse RNase L. B. Inhibition of mouse RNase L carrying indicated rat RNAse L residues (left) and of rat RNase L carrying indicated mouse RNAse L residues (right) by L*DA and L*RTV-1. Values under the lanes indicate the extent of rRNA degradation inhibition by L* compared to that mediated by L*DA on mouse RNase L (left panel) or compared to that mediated by L*RTV-1 on WT rat RNase L (right panel). (PDF) [file ppat.1006989.s002.pdf]

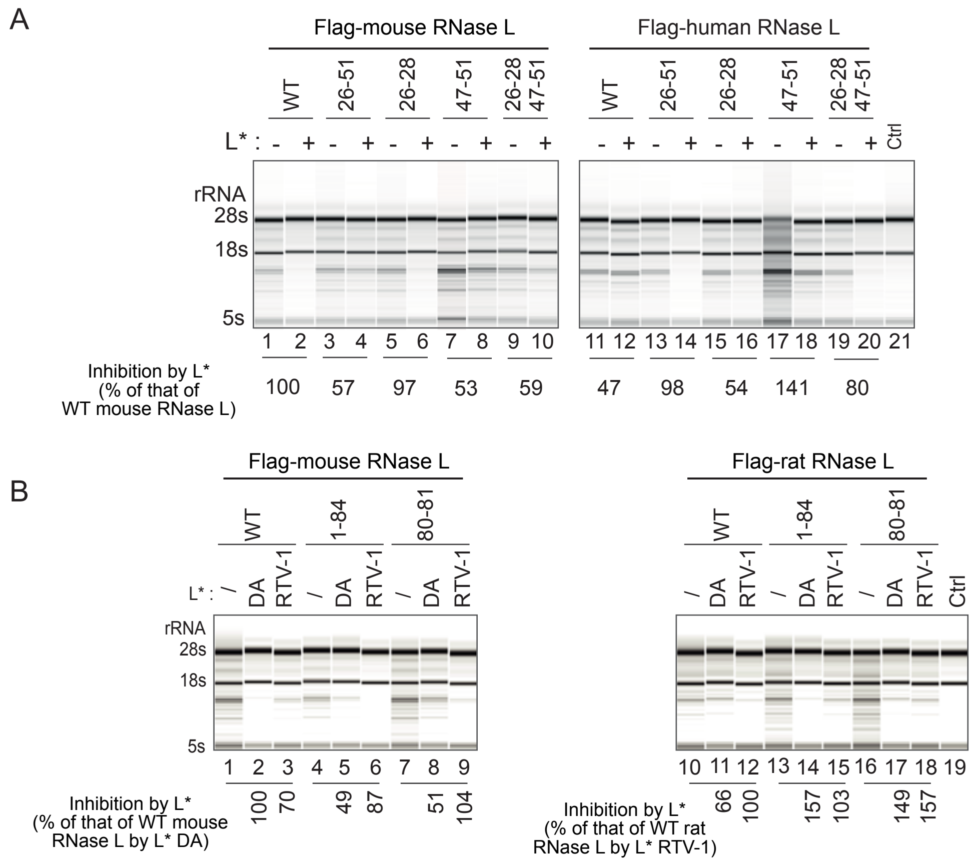

S2 Fig. Inhibition of chimeric RNase L by L*

Analysis of RNase L-mediated RNA degradation in HeLa-M cells overexpressing indicated Flag-RNase L chimera and HA-L*. RNA samples extracted 7 hours after polyI:C transfection were analyzed by RNA chips and quantified.

A. Inhibition of mouse RNase L carrying indicated human RNAse L residues (left) and of human RNase L carrying indicated mouse RNAse L residues (right) by L^*_{DA}. Values under the lanes indicate the extent of rRNA degradation inhibition compared to that of WT mouse RNase L.

B. Inhibition of mouse RNase L carrying indicated rat RNAse L residues (left) and of rat RNase L carrying indicated mouse RNAse L residues (right) by L^*_{DA} and $L^*_{RTV\text{-}1}$. Values under the lanes indicate the extent of rRNA degradation inhibition by L* compared to that mediated by L^*_{DA} on mouse RNase L (left panel) or compared to that mediated by $L^*_{RTV\text{-}1}$ on WT rat RNase L (right panel).
