# Supplementary material for: A novel mechanism of RNase L inhibition: Theiler's virus L* protein prevents 2-5A from binding to RNase L
Source: PLoS Pathog. 2018 Apr 13;14(4):e1006989. doi: 10.1371/journal.ppat.1006989 (PMC5927464; doi:10.1371/journal.ppat.1006989)
Supplement: S3 Fig — Surface plasmon resonance (SPR) experiments were performed using Biacore technology with purified recombinant L*, mouse RNase L and human RNase L. A. His6-L* was immobilized on Ni-NTA chips and human RNase L was injected at the indicated concentrations. B. His6-L* was immobilized on Ni-NTA chips and mouse RNase L was injected at the indicated concentrations. C. GST-mouse RNase L was immobilized on anti-GST CM5 sensor chip and L* was injected at the indicated concentrations. RU: response units. (PDF) [file ppat.1006989.s003.pdf]

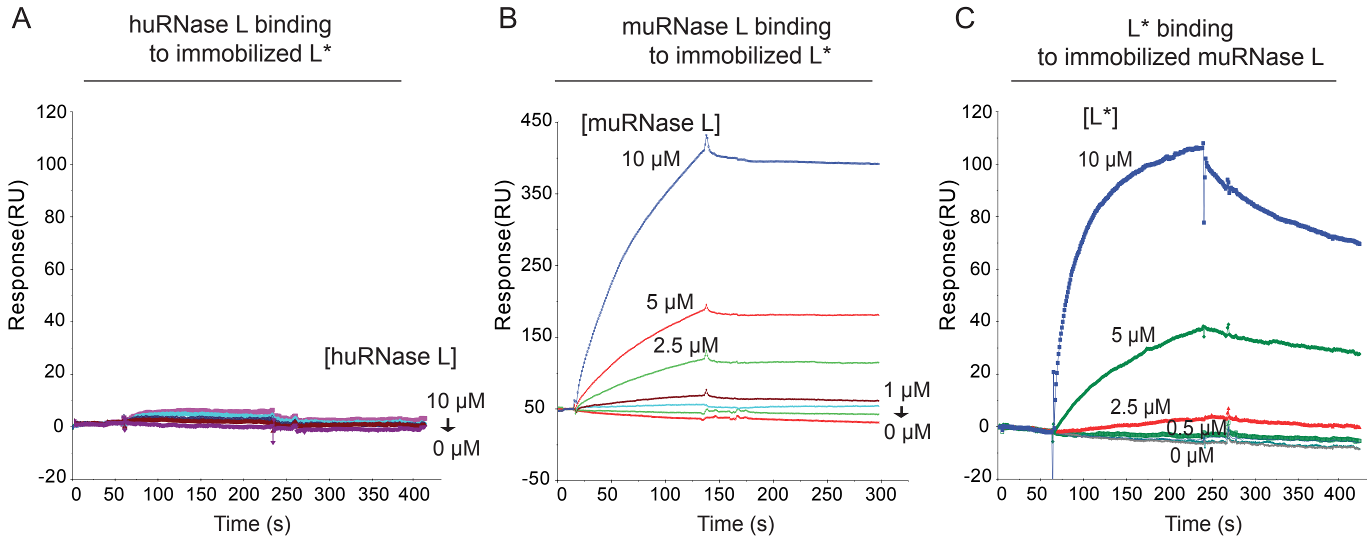

S3 Fig. Mouse RNase L but not human RNase L binds to L*.

Surface plasmon resonance (SPR) experiments were performed using Biacore technology with purified recombinant L*, mouse RNase L and human RNase L.

A. His6-L* was immobilized on Ni-NTA chips and human RNase L was injected at the indicated concentrations.

B. His6-L* was immobilized on Ni-NTA chips and mouse RNase L was injected at the indicated concentrations.

C. GST-mouse RNase L was immobilized on anti-GST CM5 sensor chip and L* was injected at the indicated concentrations.
RU: response units
