# Supplementary material for: A novel mechanism of RNase L inhibition: Theiler's virus L* protein prevents 2-5A from binding to RNase L
Source: PLoS Pathog. 2018 Apr 13;14(4):e1006989. doi: 10.1371/journal.ppat.1006989 (PMC5927464; doi:10.1371/journal.ppat.1006989)
Supplement: S4 Fig — A. Flag-L* expression in infected BMM was detected by immunoblotting using an anti-Flag antibody. B. Analysis of RNase L-mediated RNA degradation was performed by RNA chip. (PDF) [file ppat.1006989.s004.pdf]

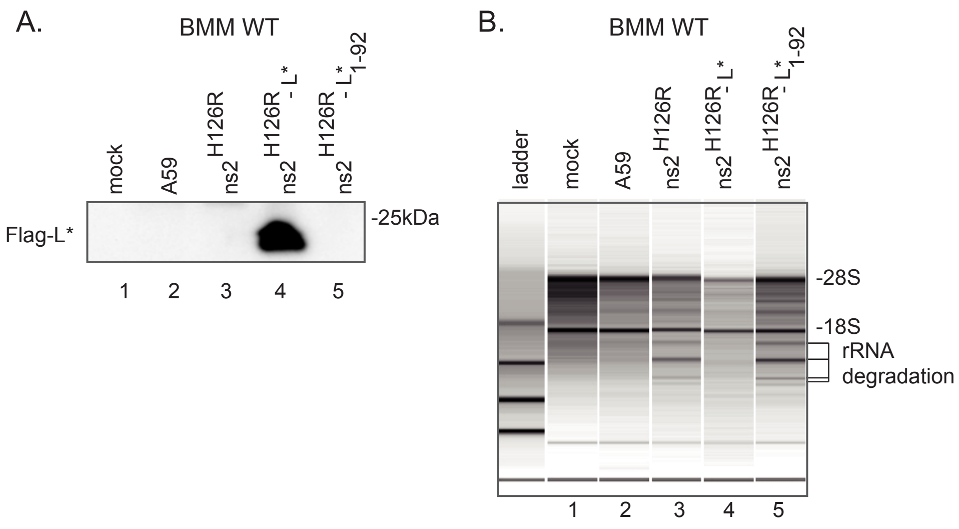

S4 Fig. Full-length L* was expressed in ns2H126R-L*-infected BMMs and inhibited RNase L-mediated RNA degradation.

A. Flag-L* expression in infected BMM detected by immunoblotting using an anti-Flag antibody

B. Analysis of RNase L-mediated RNA degradation by RNA chip.
